# Evolutionary stasis of a deep subsurface microbial lineage

**DOI:** 10.1038/s41396-021-00965-3

**Published:** 2021-04-06

**Authors:** Eric D. Becraft, Maggie C. Y. Lau Vetter, Oliver K. I. Bezuidt, Julia M. Brown, Jessica M. Labonté, Kotryna Kauneckaite-Griguole, Ruta Salkauskaite, Gediminas Alzbutas, Joshua D. Sackett, Brittany R. Kruger, Vitaly Kadnikov, Esta van Heerden, Duane Moser, Nikolai Ravin, Tullis Onstott, Ramunas Stepanauskas

**Affiliations:** 1 Bigelow Laboratory for Ocean Sciences, East Boothbay, ME USA; 2 Department of Biology, University of North Alabama, Florence, AL USA; 3 Institute of Deep-sea Science and Engineering, Chinese Academy of Sciences, Sanya, Hainan Province P. R. China; 4 Department of Geosciences, Princeton University, Princeton, NJ USA; 5 Department of Marine Biology, Texas A&M University at Galveston, Galveston, TX USA; 6 Thermo Fisher Scientific Baltics, Vilnius, Lithuania; 7 Division of Hydrologic Sciences, Desert Research Institute, Las Vegas, NV USA; 8 Institute of Bioengineering, Research Center of Biotechnology RAS, Moscow, Russia; 9 Centre for Water Sciences and Management, North West University, Potchefstroom, South Africa; 10 iWater, Bloemfontein, South Africa

**Keywords:** Environmental microbiology, Molecular evolution, Bacterial genetics

## Abstract

Sulfate-reducing bacteria *Candidatus* Desulforudis audaxviator (CDA) were originally discovered in deep fracture fluids accessed via South African gold mines and have since been found in geographically widespread deep subsurface locations. In order to constrain models for subsurface microbial evolution, we compared CDA genomes from Africa, North America and Eurasia using single cell genomics. Unexpectedly, 126 partial single amplified genomes from the three continents, a complete genome from of an isolate from Eurasia, and metagenome-assembled genomes from Africa and Eurasia shared >99.2% average nucleotide identity, low frequency of SNP’s, and near-perfectly conserved prophages and CRISPRs. Our analyses reject sample cross-contamination, recent natural dispersal, and unusually strong purifying selection as likely explanations for these unexpected results. We therefore conclude that the analyzed CDA populations underwent only minimal evolution since their physical separation, potentially as far back as the breakup of Pangea between 165 and 55 Ma ago. High-fidelity DNA replication and repair mechanisms are the most plausible explanation for the highly conserved genome of CDA. CDA presents a stark contrast to the current model organisms in microbial evolutionary studies, which often develop adaptive traits over far shorter periods of time.

## Introduction

Knowledge of mechanisms, rates and consequences of microbial evolution is critical to a wide range of scientific and practical endeavors, such as prevention and treatment of human diseases, environmental bioremediation, studies of global biogeochemical cycles and understanding the diversity of life. Our current concepts concerning microbial evolution largely rely on genome-based inferences and experimental studies applied to a small number of fast-proliferating species, primarily human pathogens and commensals [1–3]. Microbes in these laboratory-based evolutionary experiments inhabit energy- and nutrient-rich environments, have generation times ranging from minutes to days, and may accumulate nucleotide substitutions and acquire genes over the course of years or less. However, recent advances in environmental microbiology indicate that such microorganisms may be imperfect models for many bacteria and archaea, which may contain over a trillion operationally-defined lineages [4–6] and pursue widely divergent life strategies that may affect evolutionary rates.

Essentially unacknowledged until 30 years ago, deep subsurface microorganisms are now estimated to constitute ~10% of our planet’s total biomass [7, 8] and are found to contain a large fraction of yet uncharacterized biological diversity, the so-called “microbial dark matter” [6, 9–11]. Many of these microorganisms rely upon low-yield energy sources, resulting in estimated generation times ranging from months to decades [12, 13]. The Firmicute *Candidatus* Desulforudis audaxviator MP104C (CDA), originally discovered in deep continental subsurface, has emerged as one of the model microorganisms for this environment [14–17]. The CDA metagenome-assembled genome (MAG) was based upon a near-clonal population of ~10^11^ cells filtered from 5,600 liters of fracture water intersected at 2.8 km depth in the Mponeng gold mine in South Africa, where it appeared to form a single-species, chemoautotrophic ecosystem supported by H_2_, formate and sulfate that are generated in situ from radiolysis [14]. A subsequent analysis of five single amplified genomes (SAGs) of CDA relatives from 3 km deep fracture water in the neighboring Tau Tona gold mine identified novel prophages, retrons, CRISPRs, restriction-modification systems and transposases, which suggested that recombination, horizontal gene transfer (HGT) and viral infections played a significant role in the evolution of this lineage [18].

Here we report multiple SAGs of CDA from three continents: Africa (three subsurface boreholes accessed from the Mponeng, Beatrix and Tau Tona gold mines in the 3.0–2.8 Ga Witwatersrand Basin of South Africa); North America (borehole Inyo-BLM 1 (BLM1), accessing a regional fault-controlled, Paleozoic carbonate aquifer in the Death Valley Regional Flow System of southern Nevada and southeastern California); and Eurasia (borehole BY-1R, accessing a Cretaceous aquifer in the West Siberian artesian mega-basin). We also analyzed a second CDA MAG BY57 and a genome of the first laboratory culture, CDA BYF from the BY-1R site [15–17]. Given the large geographic distances separating the subsurface sampling sites, we hypothesized that CDA genomes should be genetically divergent. Further, because of the differences in the physicochemical conditions among the sampling sites, we also anticipated divergent adaptations to the local environments, i.e., that the evolutionary trajectories of the CDA populations would be analogous to those of Darwin’s finches.

## Materials and methods

### Field sample collection

**Fig. 1:**
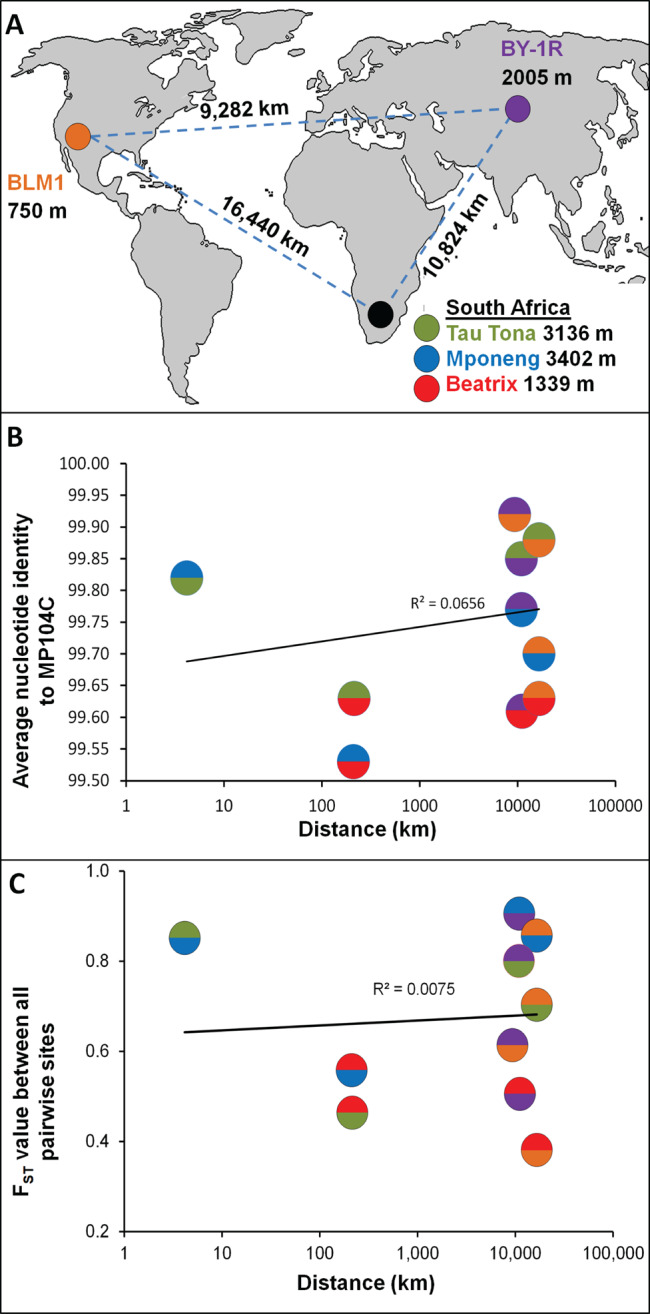
General characteristics of the analyzed *Candidatus* Desulforudis audaxviator populations. **A** Global location, distance and depth below surface of environmental samples from which CDA SAGs were obtained. **B** Average nucleotide identity in relation to geographic distance. **C** F_ST_ in relation to geographic distance. The site-specific colors of the pairwise comparisons are shown for each data point. Standard deviations and standard errors are smaller than symbols (see Table S4).

Deep fracture water was collected on January 21, 2011 from a borehole drilled at a depth of 1,339 m in Beatrix Gold Mine, on October 6, 2014 from a borehole drilled at a depth of 3,402 m in Mponeng gold mine, and on January 19, 2012 from a borehole drilled at a depth of 3,316 m in Tau Tona gold mine (Fig. 1A). The methods used in collecting and analyzing samples from mine boreholes have been previously described [19]. Mponeng and Tau Tona Mines are within ~2.7 km of each other and are 200 km from Beatrix Mine. Ground water samples from BLM1 (Fig. 1A) were collected at pressure from 755 mbls. on August 21, 2015 using a truck-mounted discrete sampler and subsampled via a sterile platinum-cured silicone hose. Physical parameters (temperature, dissolved O_2_, conductivity, and oxidation/reduction potential were obtained using an Idronaut Sonde (GeoVista, UK). The BLM1 water samples for chemistry and other measurements were collected and analyzed as described elsewhere [20]. Samples from Byelii Yar borehole 1-R (BY-1R) were collected on April 30, 2016 (Fig. 1A). Sampling and characteristics of the water chemistry have been described previously [16, 21]. All samples for SAG analyses were preserved with 5% glycerol and 1x TE buffer (final concentrations), frozen on site, and placed in a −80 °C freezer the same day upon receipt at Bigelow.

### Single cell genomics and metagenomics

The generation, identification, sequencing and de novo assembly of SAGs was performed at the Bigelow Laboratory for Ocean Sciences Single Cell Genomics Center-SCGC (scgc.bigelow.org). The cryopreserved samples were thawed, pre-screened through a 40 μm mesh size cell strainer (Becton Dickinson) and incubated with 5 µM (final concentration) SYTO-9 DNA stain (Thermo Fisher Scientific) for 10–60 min. In addition, an aliquot of the BLM1 sample was labeled with the RedoxSensor Green viability probe that detects oxidoreductase activity in sorted cells (Thermo Fisher Scientific), at a final concentration of 1 µM for 20–40 min. Fluorescence-activated cell sorting, cell size estimates, cell lysis, DNA amplification with WGA-X, sequencing (using Illumina technology), de novo genome assemblies and quality control were performed as previously described [22]. This workflow was evaluated for assembly errors using three bacterial benchmark cultures with diverse genome complexity and %GC, indicating no non-target and undefined bases and the following average frequencies of misassemblies, indels and mismatches per 100 Kbp: 1.5, 3.0, and 5.0, respectively [22]. CheckM v1.0.6 was used to calculate the estimated completeness of the SAG assemblies [23]. For SAG identification, low coverage shotgun sequencing, as well as PCR-based 16S rRNA gene screens were performed as previously described [23]. All individual SAGs were deposited in MG-RAST as site-specific CDA “metagenomes” under accession numbers mgl752158 (Beatrix), mgl752155 (Mponeng), mgl752152 (Tau Tona), mgl752164 (BLM1), and mgl752161 (BY-1R).

Metagenomic samples were collected from Beatrix gold mine 4 days after the collection of samples for single cell genomics. Microbial biomass collection, total DNA and RNA extraction, sequencing and assembly were performed as previously described [19, 24, 25]. Samples from BY-1R were collected on August 05, 2014 for metagenomic studies, and total DNA extraction, sequencing and assembly were performed as previously described [16]. Contigs representing CDA were identified and mapped to the CDA MP104C MAG using BLASTN. The circularized genome was then obtained upon joining of contigs mapped on the reference genome. Illumina reads were mapped to the CDA MP104C MAG using Bowtie 2 [26]. The correctness of the assembly was verified using Bandage [27], and by mapping metagenomic reads back to the BY-1R CDA BY57 MAG.

### Bioinformatics

The 16S rRNA gene sequences were aligned using SINA alignment software [28] and ClustalX [29]. Phylogenetic trees were inferred by MEGA 7.0 using the General TimeReversible Model, with Gamma distribution with invariable sites (G + I), and 95% partial deletion for 1000 replicate bootstraps. SAG assemblies were analyzed for protein-encoding regions using RAST (http://rast.nmpdr.org/) [30], and genes (protein families) were annotated with Koala (KEGG) [31] and InterProScan v5 [32]. Average nucleotide identity (ANI) was calculated using the online tools at the Kostas Lab website Environmental Microbial Genomics Laboratory (http://enve-omics.ce.gatech.edu) [33, 34]. SAG contig alignments to the MP104C MAG were visualized in the Geneious software suite (Biomatters, Ltd.,Auckland, New Zealand). Whole genome synteny comparisons were performed with EasyFig for Mac version 2.1 [35] with tBLASTx and the filtering of small hits and annotations option. CRISPR regions were identified using the online version of CRISPR finder [36]. QUAST was used to perform genome-wide quantification of substitutions and indels [37].

Putative phage contigs were first identified using a combination of viral marker genes (viral proteins and tRNA), DNA sequence anomalies (GC skew and tetramer frequencies), and metagenomic fragment recruitment from viral and bacterial metagenomes using methods previously described by Labonté et al. [18]. Putative viral contigs were manually inspected, and contigs that did not contain obvious phage structural genes were excluded from further analysis. Viral contigs were assembled and visualized in the Geneious software suite.

Fixation indices (F_ST_) were calculated using the ANI among SAGs as the input metric of a formula based upon Hudson et al [38]:

1 $$	F_{ST} = \\ 	\frac{{\left( {1 - 1/n\mathop {\sum}\nolimits_{i = 1}^n {{\mathrm{ANI}}\,{\mathrm{of}}\,{\mathrm{SAGs}}\,{\mathrm{between}}\,{\mathrm{sites}}}} \right) - \left( {1 - 1/m\mathop {\sum}\nolimits_{j = 1}^m {{\mathrm{ANI}}\,{\mathrm{of}}\,{\mathrm{SAGs}}\,{\mathrm{within}}\,{\mathrm{sites}}}} \right)}}{{(1 - 1/n\mathop {\sum}\nolimits_{i = 1}^n {{\mathrm{ANI}}\,{\mathrm{of}}\,{\mathrm{SAGs}}\,{\mathrm{between}}\,{\mathrm{sites}} )}}}$$

where the within and between site ANI represent averages from a pairwise matrix of all SAGs. To define pairs of protein sequences with shared similarity between the MP104C MAG and the individual 126 SAGs, an all against all BLASTP [39] search was performed using a 95% sequence identity cutoff [40]. The defined sequence pairs were subsequently aligned using Clustal Omega [41] with default parameters. Using the PAL2NAL tool [42], the nucleotide sequences that correspond with each of the aligned protein sequence pairs were converted into codon alignments. The resulting codon alignment pairs were estimated for synonymous and nonsynonymous substitution ratios using the YN00 program from PAML4.8 [43] with an implementation of the Yang and Nielsen 2000 method [44]. Synonymous and nonsynonymous substitution ratios were also calculated for two monocultures of *Prochlorococcus marinus* MIT9313 [45] and *Synechococcus* sp. WH8102 SAGs [46] relative to their original sequenced genomes, and current SAG co-assemblies. The finished genomes for these two strains are similar in size (2.41 and 2.64 Mbp) to that of CDA MP104C MAG (2.35 Mbp). In parallel to these, a similar approach was repeated for the 7 *Sulfolobus islandicus* genomes [47], where pairwise estimations of synonymous and nonsynonymous substitution ratios were conducted for all genome pairs, instead of each relative to a reference. DNA polymerase modeling and fidelity testing methods are described in the Supplemental Material.

## Results

### Global conservation of CDA genomes

Of the 150 CDA SAGs obtained in this study, 136 yielded a 16S rRNA gene 100% identical to that of the CDA MP104C MAG, whereas 14 had 97–99% 16S rRNA gene identity. Of these 136 CDA SAGs, 126 produced >100 kbp assemblies and were analyzed further (Table S1). The estimated completeness of the individual SAG assemblies was <1–67%, with no indications of contamination. The Mponeng 120 fracture water contained only CDA SAGs, but was collected from a different fracture, located 1.3 km to the southeast of the fracture that yielded CDA MP104C, and was lower in salinity than that of the MP104C fracture water [14]. The remaining four sites had relatively simple microbiomes, where CDA ranged from 10 to 40% of the SAGs (Fig. S1).

**Fig. 2:**
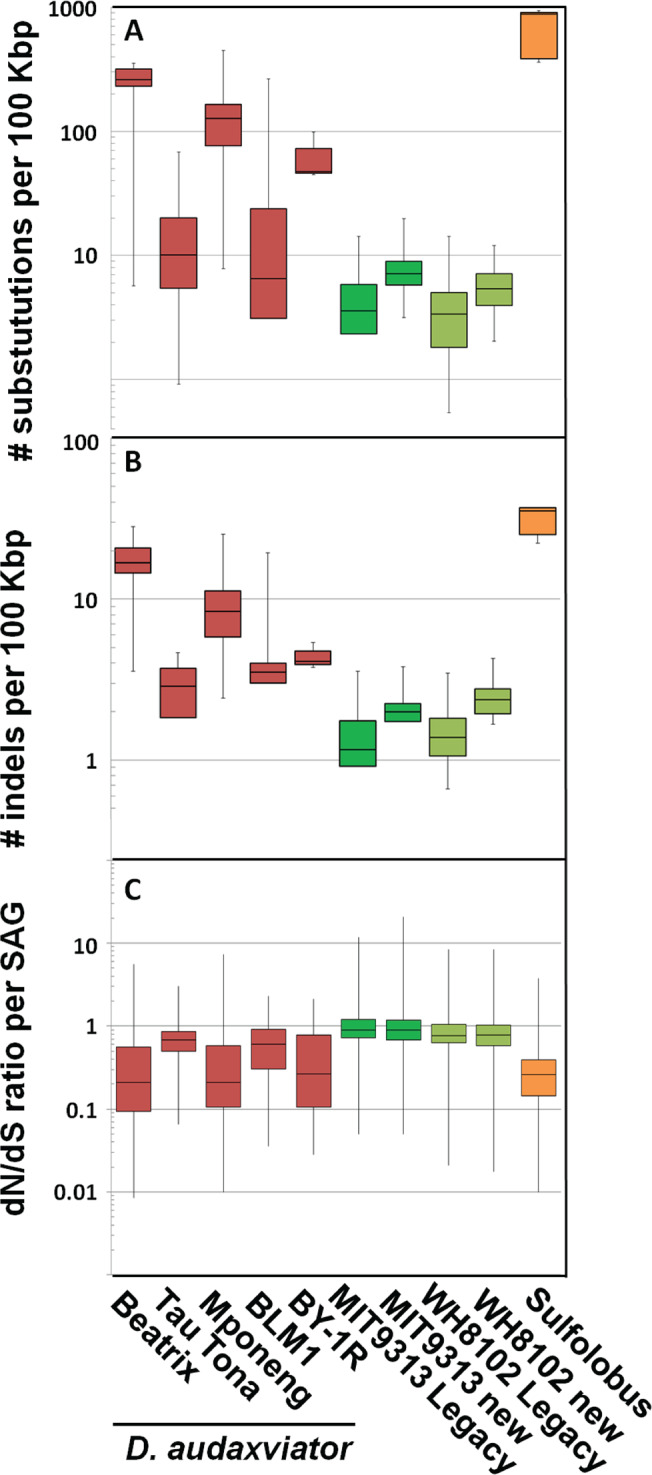
Evolutionary analyses of *Candidatus* Desulforudis audaxviator (CDA) SAGs. Genomic variability among *Candidatus* Desulforudis audaxviator SAGs (red); SAGs of cyanobacteria cultures MIT9313 and WH8102 (green); and *Sulfolobus islandicus* genomes (orange) [47]. Variability metrics include: (**A**) nucleotide substitutions; (**B**) indels; and (**C**) dN/dS ratio, with respect to the MP104C reference genome. CDA SAGs are separated by study site. Cyanobacteria values, which were used as methodological controls, were calculated using two references: legacy genome sequences [45, 46] and new co-assemblies of SAGs.

Despite the fact that the 126 CDA SAGs were obtained from North American, Eurasian and South African sites (Fig. 1A) with distinct microbial communities (Fig. S1) and physicochemical environments (e.g., temperature 37–65 °C; Eh −89 to −337 mV; pH 6.9–8.8; and TDS 0.3–4.5 ppt; Table S2), the genomes of these SAGs were nearly identical to each other and to the CDA MP104C MAG. The ANI of all pairwise comparisons exceeded 99.5% and did not correlate with geographic distance (Fig. 1B, Table S3). An average of 94% of SAG base pairs aligned to the MP104C MAG (Table S3), with prophage regions and unique genes (e.g., *phn* operon) accounting for the bulk of the unaligned regions. These results are in good agreement with the limited genomic differences between CDA MP104C MAG and the isolate CDA BYF from the West Siberian borehole (ANI of 99.5%) [17], and SAG pairwise ANI’s compared to CDA BYF ( > 99.8%). In comparison to the CDA MP104C MAG, the average nucleotide substitutions and indels of the sequenced CDA SAGs ranged from <1 to 448 and <1 to 28 per 100 Kbps, respectively (Fig. 2A, B) and did not correlate with CDA SAG completeness (Fig. S2). Although the number of substitutions and indels per 100 Kbps in CDA SAGs were low, they were higher than the corresponding values for MIT9313 and WH8102 monocultures (*p* < 0.05, ANOVA), suggesting that they were above the methodological detection limits (Fig. 2A, B). These findings stand in stark contrast to the extensive genome differences among isolates of well-characterized model organisms [48] or marine bacterioplankton in a single drop of sea water [49, 50]. Interestingly, despite the extremely high ANI among all CDA SAGs, ANI values were higher within CDA populations than across populations, with no relationship to geographic distance (Fig. 1C, Table S4).

### Evidence of HGT and phage/CRISPR conservation

We found 21 genes unique to a particular study site, often showing partial homology to other microorganisms from the subsurface (Table S5). For example, multiple CDA SAGs from Mponeng encoded an arsenite transporter, a permease, and an entire phosphonate uptake and utilization operon (*phnCDEGHIJKM*), which were absent in other study sites. Interestingly, *phnDEIMJ* had 33–70% ANI to Betaproteobacteria *Thiobacillus denitrificans*, while *phnCGHK* had 40–50% ANI to a Firmicutes Peptococcaceae lineage, both of which were found among SAGs from the nearby Tau Tona site (Fig. S3). Mponeng CDA SAGs also contained homologs of *phnE*, two of which co-localized with ABC phosphonate transporters. One *phnE* gene had 47% ANI to a Bacteriodetes lineage (Fig. S3). One CDA SAG from Beatrix and one CDA SAG from Tau Tona contained a GDP-mannose 4,6-dehydratase (*gmd*) gene with 85% ANI to a Nitrospirae genome from BLM1. These findings suggest that HGT and recombination, although infrequent, played a role in the slight genomic divergence of CDA populations from the three continents.

**Fig. 3:**
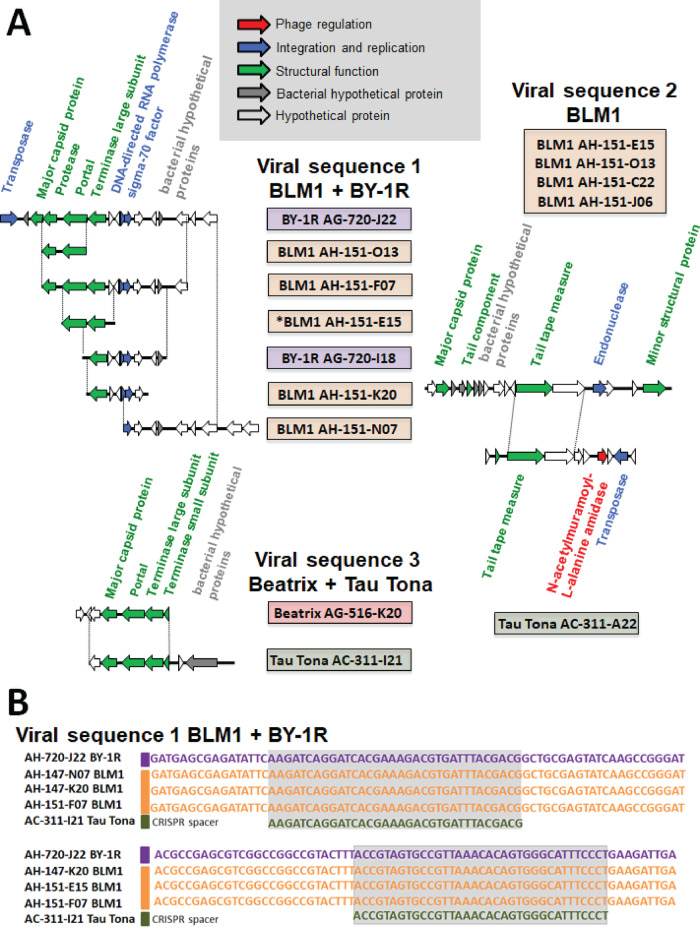
Examples of identical prophage and CRISPR sequences in SAGs from geographically distant locations (see Table S6 for a complete list). **A** Alignments of SAG prophages sourced from multiple field sites. Viral sequence 1 was found in SAGs from BLM1 (North America) and BY-1R (Eurasia). Viral sequence 2 was found in SAGs from BLM1 (North America) and Tau Tona (Africa). Viral sequence 3 was found in SAGs from Beatrix (Africa) and Tau Tona. Dotted lines show regions with 100% nucleotide identity. Asterisk in BLM1 AH-151-E15 represents the one single nucleotide insert in the alignments. SAGs are color-coded by site; BY-1R, purple; BLM1, orange; Tau Tona, green; and Beatrix, red. **B** Prophage contigs from BLM1 (North America) and BY-1R (Eurasia) aligned to two different CRISPR spacer regions from Tau Tona (Africa).

We found 25 regions with phage marker genes in 23 CDA SAGs from Mponeng, BLM1, BY-R1 and Tau Tona. Of those, 10 contigs from different sites shared 100% nucleotide identity in overlapping regions with the exception of one nucleotide insert (Fig. 3A and Table S6). The presence of bacterial genes flanking at least one putative prophage region (Fig. 3A; gray arrows), the failure to recover complete phage genomes from the SAGs, and the lack of anomalies in the relationship between single cell whole genome amplification speed versus host genome recovery (Fig. S4) all suggest lysogeny rather than lytic infections, in agreement with earlier findings from the Tau Tona CDA SAGs [18].

We also identified 33 partial CRISPR regions and associated proteins, collectively containing 138 unique spacer regions across all CDA SAGs with 100% nucleotide identity. This is consistent with the comparison of the CRISPR regions of the CDA BYF genome and CDA MP104C MAG, which revealed near-complete conservation of CRISPR region order and nucleotide-level identity, with the exception of one SNP and five additional spacer regions found along the length of the array [17]. Additional spacers were not located toward the beginning of the array, where they would be inserted in response to recent viral infections [17]. CRISPR repeats and spacer regions identified in CDA MP104C MAG were also found in CDA SAGs from all three continents. Two CRISPR spacers in Tau Tona CDA SAGs had 100% sequence identity to five putative prophages in CDA SAGs from BLM1 and the CDA BYF genome (Fig. 3C). No prophage-like sequences matching this spacer were identified in Tau Tona, which is expected if the CRISPR spacer prevents infection from the corresponding phage.

## Discussion

### Global dispersal

CDA genomes from all three continents revealed a striking degree of conservation manifested in high ANI, few SNP’s, and the conservation of prophages and CRISPRs. The latter is particularly surprising, since CRISPRs are generally considered one of the most rapidly evolving genome regions [51]. This prompted us to consider the following possible mechanisms to explain these observations: (1) cross-contamination of CDA in the lab or in the field; (2) recent dispersal between the subsurface sites via aerial transport; and (3) ancient dispersal combined with evolutionary stasis.

SAG analyses were performed in a cleanroom environment that has been consistently proven to prevent sample cross-contamination [22, 52], and CDA SAG sample analyses were separated by several years. For example, the CDA SAG samples from Tau Tona that contain viral contigs and CRISPR sequences identical to viral elements in CDA SAG samples from BLM1 and BY-R1 were sorted, amplified and sequenced in 2012, whereas those from BLM1 and BY-R1 were analyzed in 2016 and 2017, respectively. The sample containing the CDA MP104C MAG, which has CRISPR elements identical to those of the BLM1 SAGs and CDA BYF, was collected from a freshly drilled borehole in 2002, DNA was extracted in 2003 at the Princeton University lab and the metagenome sequenced at the Joint Genome Institute (JGI) in 2005. The CDA SAG samples did not arrive at the Princeton University lab until 2012–2014 and were shipped directly to SCGC upon arrival without opening. Even if one speculates that the CDA SAGs were cross-contaminated, there is no way to explain the 100% identity in CRISPR elements between the CDA MP104C MAG sequenced at JGI in the U.S. in 2005 and the CDA BYF culture isolated and sequenced in Russia in 2018 as cross-contamination.

Next, we examined the possibility of anthropogenic cross-contamination in the field. To the best of our knowledge, there has never been a cross-use of drilling or sampling equipment among our South African mine drilling contractors, the U.S. Department of Energy well drilling contractors for BLM1 completed in 2007, and Russian oil well drilling contractors for the Byelii Yar oil well, which was drilled in 1962. In South Africa, CDA has been detected in many deep ground water and fracture water samples across the Witwatersrand Basin [53–55] in boreholes drilled between the mid-1990’s and 2011, but it has never been found in water used for mining operations [54]. Likewise, investigators involved in the collection of the Siberian sample never visited our sites in South Africa and California, and vice versa. Only one investigator who collected samples from South African sites also did so from the Californian site, but after a 17-year interlude. We conclude that sampling cross-contamination cannot explain the high degree of genome similarity among the analyzed CDA populations on three continents.

We assessed the possibility of recent natural dispersal as an explanation for CDA genome conservation. For instance, *Sulfolobus islandicus*, *Sulfolobus acidocaldaria* and *Thermus* species from globally distributed hot springs revealed similar genomes, but contained from 10× to 100× higher substitution frequencies and indels [47, 56–58] than CDA genomes. *Sulfolobus acidocaldaria* and *Thermus* CRISPR spacers were found to be mostly conserved across multiple continents, though the number of shared spacers decreased with increasing geographic distance [58], unlike those of the CDA genomes. If the *Sulfolobus islandicus* genome SNP’s were acquired over an estimated 910,000 year period since population separation by aerial dispersal, which is based upon the ages of the volcanic formations, then the average rate of nucleotide substitution is a minimum of 4.7 × 10^−9^ substitutions per site per year [47]. Using this rate, Karnachuk et al. [17] estimated a divergence time of ~2800 to 3.1 million years for the MP104C MAG and CDA BYF genome. To fit this time frame, Karnachuk et al. [17] hypothesized that CDA were dispersed aerially as spores. Although CDA BYF does exhibit some short term tolerance of microaerophilic conditions [17], CDA lacks O_2_ protection genes [14], suggesting that frequent, long-distance migration over surface environments (e.g., via air and water) in vegetative form is unlikely.

For additional evidence of CDA global dispersal by air, we searched for 16S rRNA gene sequences with ≥99% identity to the MP104C MAG in public databases: NCBI Genbank, RDP II [59], SILVA [28], Greengenes [60], the JGI Integrated Microbial Genomes database [61], Integrated Microbial Next Generation Sequencing (IMNGS) [62], iMicrobe [63], the Earth Microbiome Project [64] and Tara Ocean Project [65]. Most commonly used PCR primers for bacterial 16S rRNA genes have high estimated binding efficiency for CDA, and only a less commonly used primer 341-f had mismatches (Table S7). Excluding the sites reported in this study, we found only 1854 partial 16S rRNA sequences with ≥99% nucleotide identity to CDA in all IMNGS metagenomes and other datasets. With the exception of several reads present in seafloor sediments near a methane seep off the coast of Oregon [66] and one read in Guaymas Basin sediment [67], all reads were found in samples from continental or island subsurface sites 400–1200 m deep (Table S8). Most of the reads originated from a Pleistocene basaltic aquifer in Iceland [68, 69]; an additional location in the same Paleozoic dolomite aquifer accessed by BLM1 and in the overlying Miocene volcanic units in Nevada [20]; in Neogene-Paleogene and Cretaceous gas reservoirs in Japan [70, 71]; and in Upper Cretaceous coal bed gas reservoirs in Alberta, Canada [72, 73]. A single CDA sequence read originated from a biofilm in an anaerobic geothermal reactor in Denmark; which could have originated from subsurface geothermal water that supplied the facility [74]. CDA was not detected in any hot springs, including those in South Africa [75]. Even though a few CDA-like 16 S rRNA sequences were detected at one marine methane seep [66], deposition at our mid-continent sites would still require aerial transport, which should leave a footprint in soil sites, but no CDA have been reported in soils. Given the large scale of current 16S rRNA gene surveys, in particular the Earth Microbiome project that encompasses 2.2 billion 16S rRNA gene sequences from 27,751 environmentally diverse and globally distributed samples, and the tens of thousands of environmental datasets in IMNGS, our findings suggest no current dispersal of CDA by air. The one caveat to this evidence is that aerial dispersal could have occurred tens to hundreds of thousands of years ago and would be undetectable today given the survival time of DNA in soil [76]. Two other arguments oppose the aerial dispersal as spores; (1) the 2800–3.1-million-year separation age is younger than the 20 myr old ground water ages for some of the CDA-bearing South African sites (Fig. S5); and (2) thermophilic spore lifetimes are on the order of hundreds of years [77, 78], which is far shorter than the ground water ages for the CDA-bearing South African sites, implying that CDA spores would be dead before reaching subsurface fractures to germinate.

The final possibility is that CDA has been dispersed in the subsurface via migration along ground water flow paths in a primarily vegetative state. This is unlikely, given CDA cannot grow at sea water salinity [17] and has not been reported in deep sea vents [79]. CDA is distributed over a distance of at least 300 km in the South African Witwatersrand Basin by fluid flow, primarily along fractures, and likely has been residing there for at least 20 myr (Fig. S5). However, CDA, like all subsurface microorganisms, likely spend most of their time attached to mineral surfaces, perhaps in microcolonies, and the dispersal times across even thousands of kilometers may require tens to hundreds of millions of years.

F_ST_ is a metric commonly used in ecology to measure genetic exchange between eukaryotic populations. Although the CDA populations are genetically very similar (Table S4 and Fig. S6), F_ST_ values demonstrated they are genetically divergent from one another (Fig. 1C). We are not aware of other F_ST_ analyses of natural microbial populations, but F_ST_ values of >0.9 suggest a very limited exchange between CDA populations after separation [38].

A minimum time for separation of the CDA habitats from our study sites is set by the ~165 Ma separation time for Africa from Laurentia (North America + Eurasia), with the formation of the Central Atlantic rift zone [80], and the ~55 Ma separation time for North America from Eurasia with the formation of the North Atlantic rift zone [81]. At ~47 Ma, the Jan Mayen Microcontinent broke away from Greenland and was buried beneath the Icelandic Plateau [81]. Japan was connected to the Asian continent at the time the CDA-bearing sediments were deposited, and did not split from Asia until ~15 Ma [82]. Although the separation of our CDA populations could have occurred during the breakup of supercontinents prior to Gondwana, even this most recent separation time requires remarkable genome stability.

### Potential drivers of genome stability

**Fig. 4:**
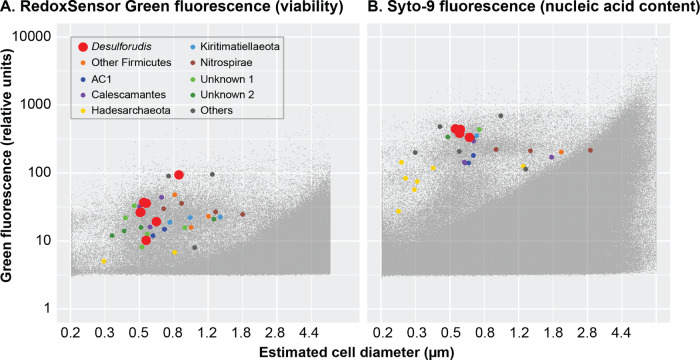
Flow-cytometric characterization of microorganisms from BLM1. **A** Oxidoreductase activity and (**B**) nucleic acid content analyzed by flow cytometry at BLM1 site in California. Identified cells are color-coded, with CDA colored red and enlarged for visual recognition. The *Y*-axis is fluorescence. The *X*-axis is estimated diameter of the sorted cells, derived from the forward light scatter.

In order to evaluate the potential mechanisms leading to the unusual genome stability of CDA, we considered dormancy as a spore [14, 17], purifying selection and high-fidelity DNA replication and repair mechanisms. Scanning electron microscopy demonstrated the predominance of vegetative cells in the MP104C MAG sample, which contained only CDA [14], while metatranscriptome analyses of the Beatrix fracture water [19] indicated that CDA were actively expressing sulfate reducing genes (Table S9). The in situ turnover time of CDA biomass at the MP104C MAG site was estimated from aspartic acid racemization at <1 year [13]. Analyses of the CDA BYF isolate revealed it rarely formed spores [17]. In this study, we found that CDA from BLM1 expressed oxidoreductase activity and readily stained with a nucleic acid dye (Fig. 4), providing further support for their in situ vegetative state. We were unable to analyze oxidoreductase activity for the South African and BYR-1 samples, as the protocol was not in place when the samples were processed. Recent data show that spore lifetimes of thermophiles are not significantly longer than those of vegetative cells under starvation conditions at optimal growth temperatures [77]. In addition, due to the amino acid racemization-induced mortality [77], dormancy extending beyond a few years at thermophilic temperatures is unlikely [13]. The collective evidence from the various techniques suggests that the studied CDA populations contained metabolically active and replicating cells, which, depending upon the temperature, suggests doubling times of no more than <1–10 years.

Next, we considered the possibility of selective forces playing a role in the conservation of CDA genomes. The genome-wide ratio of non-synonymous to synonymous substitutions (dN/dS) averaged between 0.21 and 0.68 in the studied populations (Fig. 2C). These values are higher than in most other microbial populations hypothesized to be under purifying selection, such as marine *Marinimicrobia* (<0.1) [83], Cyanobacteria (0.03–0.08) [84] and Alphaproteobacteria (0.00–0.25) [85], and diverse Gammaproteobacteria (average of 0.05) [86]. We conclude that the relatively high dN/dS of the analyzed CDA populations provides no evidence for unusually strong purifying selection in this lineage.

**Fig. 5:**
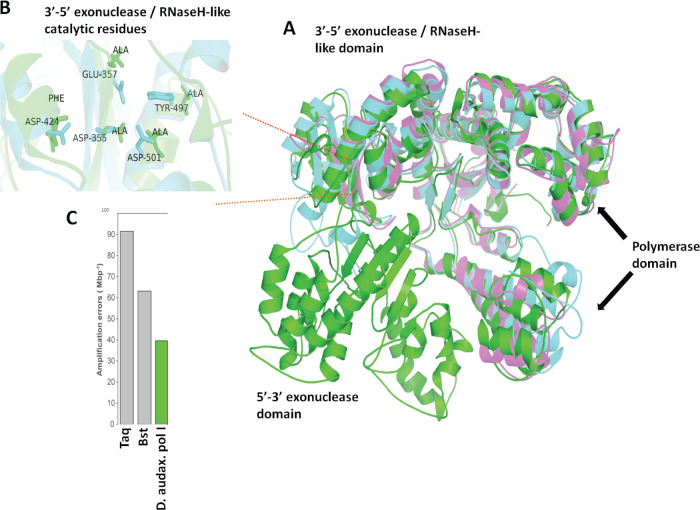
*Candidatus* Desulforudis audaxviator DNA polymerase I model. **A** Models of the large fragment of polymerase I of CDA (green), large Klenow fragment of *E. coli* DNA polymerase I (cyan) and *Thermus aquaticus* DNA polymerase I (purple). See Tables S10A–C for corresponding protein and domain IDs. **B** Enlarged view of the 3′-5′ exonuclease site and RNaseH-like catalytic residues. **C** Experimental evaluation of the fidelity of CDA DNA polymerase I, in comparison to Taq and Bst polymerases.

We examined DNA repair mechanisms and polymerase fidelity as factors potentially contributing to genome stability (Table S10). CDA SAGs and the MP104C MAG encode at least seven DNA repair mechanisms: MutL-MutS system, UvrABC system, DinG, RecA, RadA, RecFOR, RecBCD, UvrD; as well as other nucleases involved in end-joining, internal excision, and end trimming. Furthermore, CDA encode DNA-binding proteins (e.g., HU-beta), which may also reduce mutation rates [87, 88]. CDA genomes encode DNA polymerases I (*pol*), III, IV, and X. The *polymerase* I of CDA contains multiple non-overlapping domains (starting from N-terminus): 5′-3′ exonuclease domain, Ribonuclease H superfamily domain, and palm domain of family A DNA polymerase (Fig. 5A, B). The Ribonuclease H superfamily domain is responsible for the 3′-5′ proofreading exonuclease activity in *E. coli*, whereas it is inactive in *Thermus aquaticus* [89]. Structural analysis, however, indicated that the CDA polymerase I is unlikely to possess 3′-5′ proofreading activity (Figs. S7 and S8).

In order to experimentally evaluate the fidelity of CDA DNA polymerases I, IV and X, we synthesized them by heterologous expression in *E. coli*. Only polymerase I was obtained in a water-soluble form and could be analyzed further. This polymerase phylogenetically clusters with a mixed group of *Thermoanaerobacterales* and deeply branching *Clostridiales*, with the closest ortholog being from *Desulfovirgula thermocuniculi* (Thermoanaerobacterales) (Fig. S9). Under the applied in vitro conditions, this polymerase produced 40 mismatches per one Mbp, which demonstrates higher fidelity than Taq and Bst (Fig. 5C), but lower than some of the enzymes utilized in isothermal DNA amplification, such as phi29 [90]. However, the hypothesized high fidelity of CDA DNA replication could still be a result of factors other than polymerase I, such as highly accurate DNA polymerases IV and X or sophisticated DNA repair mechanisms.

## Concluding remarks

The collective evidence suggests that minimal evolution has taken place in the studied CDA populations from African, Eurasian and North American sites since their separation from the ancestral population. Based upon our analyses, the most likely scenario is that this separation occurred between 165 and 55 Ma, during the breakup of Pangea. High fidelity of DNA replication and repair mechanisms remains the most plausible mechanisms behind this extreme genome conservation, although we were unable to confirm this in vitro.

CDA presents a stark contrast to the current model organisms in microbial evolutionary studies, which are found to develop adaptive traits over far shorter periods of time [2]. Our findings suggest that the separated CDA populations are more analogous to Darwin’s finches with subtle variations in color as opposed to large differences in beak size, and call for a re-evaluation of some of the explicit and implicit assumptions about microbial evolution. For example, long, unknown periods of evolutionary stasis may impact the scaling of the molecular clock [1], the topology of inferred phylogenetic relationships, and the development of standardized divergence thresholds for a streamlined microbial taxonomy [91, 92]. The hypothesized high fidelity of CDA DNA replication and repair mechanisms may find practical applications in biotechnology. Since the subsurface harbors an estimated 10% of planet’s microbial biomass and a large fraction of its biodiversity, it may be expected that CDA is not the only living microbial fossil in this vast environment, potentially offering unique sources of information about the history of life.

## Supplementary information

Supplementary Materials for the article entitled: Evolutionary stasis of a deep subsurface microbial lineage
