# Supplementary material for: Evolutionary stasis of a deep subsurface microbial lineage
Source: ISME J. 2021 Apr 6;15(10):2830–42. doi: 10.1038/s41396-021-00965-3 (PMC8443664; doi:10.1038/s41396-021-00965-3)
Supplement: Supplementary file 1 — Supplementary Materials for the article entitled: Evolutionary stasis of a deep subsurface microbial lineage [file 41396_2021_965_MOESM1_ESM.pdf]

Supplementary Materials for the article entitled: Evolutionary stasis of a deep subsurface microbial lineage

Eric D. Becraft, Maggie C.Y. Lau Vetter, Oliver K.I. Bezuidt, Julia M. Brown, Jessica M. Labonté, Kotryna Kauneckaite-Griguole, Ruta Salkauskaite, Gediminas Alzbutas, Joshua D. Sackett, Brittany R. Kruger, Vitaly Kadnikov, Esta van Heerden, Duane Moser, Nikolai Ravin, Tullis Onstott, and Ramunas Stepanauskas

Correspondence to: rstepanauskas@bigelow.org

This file includes:

Extended Materials and Methods

Tables S1-S10

Figures S1-S9

References

Field Sites and Geological Context

Africa. The Witwatersrand Basin lies within the Archaean Kaapvaal Craton of South Africa and is comprised of three major stratigraphic units from bottom to top, the 2.9-Ga quartzites of the Witwatersrand Supergroup, the 2.7-Ga metamorphosed basalt and basaltic andesite of the Ventersdorp Supergroup, and the chemical sediments and volcanic strata of the 2.5-Ga Transvaal Supergroup [1]. The latter overlie the former and all overlie a 3.0-Ga granitic basement. The Transvaal Supergroup represents the last marine transgression in this region of the Kaapvaal Craton.

Fracture water samples were obtained from three gold mines in two gold mining districts of the Witwatersrand Basin. Beatrix Gold Mine (28.240S, 26.796E, 1,375 meters above sea level, masl.) of Sibanye Gold Limited lies in the Welkom district, located in the southwest portion of the basin. Here, the Witwatersrand and Ventersdorp Supergroups are overlain by 600 m of Carboniferous to Jurassic Karoo sediments [2]. Water samples from here were taken from a borehole into the Central Rand Group of the Witwatersrand Supergroup at 1,339 m depth [3, 4]. Tau Tona and Mponeng gold mines of AngloGold Ashanti Limited lie in the northwestern rim of the Witwatersrand Basin in the Carletonville mining district, where the Witwatersrand and Ventersdorp Supergroups are overlain by the dolomite aquifer of the Transvaal Supergroup. At Mponeng gold mine (26.448S, 27.442, 1,600 masl.), water samples were taken from a borehole into the Central Rand Group of the Witwatersrand Supergroup at 3,420 m depth, while at the Tau Tona gold mine (26.147S, 27.427E, 1,658 masl.) water samples were collected from a borehole into the Central Rand Group of the Witwatersrand Supergroup at 3,140 m depth.

Eurasia (Siberia). The oil exploration borehole 1-R, drilled in 1961-1962 to 2,563 m depth, is located in the town Byelii Yar in Tomsk region, Russia (58.4496N, 85.0279E, 84 masl.), and has been previously described in detail [5]. The Western Siberian artesian basin is a synclinal structure containing Mesozoic, Tertiary and Quaternary sediments, overlaying a Paleozoic basement. This region is known as one of the world's largest oil reserves formed from marine sediments of the Mesozoic Era. The borehole penetrated about 25 m of Quaternary sediments followed by ~150 m of Paleogene sediments and Cretaceous sediments down to 2,186 m depth [6]. Upon passing Jurassic sediments, at 2,505 m the borehole entered a Paleozoic basement, comprising sedimentary rocks up to 2,534 m and then basalts [6]. Upon completion, the borehole was tested by a bottom-up casing perforation method. The water flowing out of the borehole is expected to originate from an aquifer system located at 1,997 to 2,005 m depth interval in the early Cretaceous sedimentary rocks (150-100 Ma).

The sampled water was slightly alkaline (pH 7.9 to 8.5) and highly reduced (Eh from 341 – 279 mV). Ionic content of the water is dominated by sodium as a cation, and chloride as an anion, with a moderate bicarbonate alkalinity. The Cl/Br mass ratio was about 270, a value close to seawater (290), suggesting that the water salinity is likely to be derived from marine salts of a relict ocean. The total mineralization of the water was 1.8 g L^{-1}, accounting for only 5% of marine salinity. Therefore, most of the water is likely derived from meteoric recharge. This is consistent with the signatures of stable isotopes ^{18}O and ^{2}H, which were similar but not identical to meteoric water [6]. Magnesium/strontium mass ratio was very low (0.14), suggesting that strontium accumulated in water during prolonged residence time, while the magnesium was precipitated presumably by dolomitization. Another evidence of a prolonged residence time is a high

concentration of fluoride that may have resulted from the dissolution of fluoride-containing minerals.

North America. Monitoring well Inyo-BLM 1 (36.4004N, -116.4692W, 694 masl.) is located in the Death Valley Regional Flow System (DVRFS) of the Basin and Range (B&R) physiographic province of the western United States. Paleozoic sedimentary rocks that host the DVRFS carbonate aquifer system underwent tectonic extension during the Tertiary, resulting in numerous low-angle faults [7]. Inyo-BLM 1 samples the "Lower Carbonate Aquifer [LCA]") of the DVRFS. The LCA is recharged via secondary fracture permeability and "interbasin flow" by groundwater associated with montane zones 10 to >100 km to the North and East, ultimately discharging to the Death Valley salt pan at ~86 masl, the lowest point in North America [8, 9]. Inyo-BLM 1 was drilled and completed to a depth of 871 m in 2007 by The Hydrodynamics Group, LLC. (Redmond, WA) for Inyo County, California. It intersects lithologies, ranging from lake sediments and volcanic tuff, to valley fill alluvium and ultimately the Hidden Valley Dolomite at ~748 mbls. The well is continuously cased in low-carbon steel to a depth of 750 mbls. to isolate the shallower Tertiary aquifers from the LCA and was sited, based on geophysical measurements, to target an inferred buried carbonate pinnacle hydrologically connected to a deeper carbonate mass [7].

Age estimation of sample sites

The South African fracture waters encountered at 1 to 3.4 km depth in the Witwatersrand Basin gold mines represent mixtures of paleometeoric water and 2.0 Ga hydrothermal fluids [11, 12]. The 1.4 km deep brackish fracture water from Beatrix gold mine have yielded bulk noble gas isotope, subsurface isolation ages of 4.3±0.7 Myrs, which is consistent with ^{36}Cl analyses [12],

although ^{14}C analyses of the dissolved inorganic carbon (DIC) suggest that a small fraction of fresh water that is <20-32 Kyr has mixed with it [13]. The 3.1 km deep fresh fracture water from Tau Tona Gold Mine has yielded bulk noble gas isotope, subsurface isolation ages of 0.7±0.5 Myr [14] and ^{14}C DIC ages of 16.3-21.9 Kyr [13]. The 3.4 km deep brackish fracture water from Mponeng Gold Mine has not been dated. However, its chlorinity, when compared to that of other fracture waters from the Witwatersrand Basin gold mines that have been dated (Fig. S5), suggests a subsurface isolation time of between 30 Kyr and 4 Myr. It is important to note that the Mponeng borehole sampled in this study is approximately 1.3 km southeast of the borehole from which the fracture water sample for the MP104C assembly was retrieved [15, 16].

The North American site Inyo-BLM 1 borehole accesses brackish meteoric water from 752 meters depth. The comparatively high temperature (57°C) at shallow depth and elevated Na^+, K^+, SO_4^{2-}, Cl^- in Inyo-BLM 1 (Table S2, 10) indicates mixing with a deeper and likely older volcanic-influenced source [17]. This mixing model is supported by multi-fraction ^{14}C analysis of Inyo-BLM 1 water, which shows a DIC age of 34.0±0.4 Kyr (likely representing some degree of equilibrium with host rock), a dissolved organic carbon (DOC) age of 4.42±0.03 Kyr (suggestive of paleometeoric recharge water contribution), and a particulate organic carbon (POC) age of 18.05 ±0.11 Kyr (Fig. S5; unpublished data).

The BY-1R borehole accesses brackish water from 2 km depth, which represents a mixture of modern meteoric water and up to 5% saline water that may represent a relic of Cretaceous sea water [6]. Neither cosmogenic nor noble gas isotope analyses to date the water from borehole BY-1R have been conducted.

Modeling of DNA polymerase I

DNA polymerases and DNA repair proteins were identified using InterProScan (Table S10A). Domains in *D. audaxivator* DNA polymerase I (UniProt ID: B1I4C0_DESAP) were identified using InterProScan server (accessed August 2020) [18, 19] and compared to reference proteins deposited in the Uniprot database [19] (Table S10B). Structure modelling was done using I-TASSER (PMID: 20360767, 25549265, 25883148). Modelling was done for the full-length protein and for a region matching the RNaseH-like domain. The closest structural analogs were identified by TM-align (PMID: 15849316). 2FKZ (chain A) and 5KTQ (chain A) structures were superimposed with resulting full length protein model and visualized using PyMol [21]. **I**dentification of potential catalytic residues at 3'-5' exonuclease / RNaseH-like domain was done based on sequence alignment produced with PROMALS3D (PMID: 18287115).

The *polymerase* I of CDA contains multiple non-overlapping domains (starting from N-terminus): 5'-3' exonuclease domain, Ribonuclease H superfamily domain and palm domain of family A DNA polymerase. The Ribonuclease H superfamily domain is responsible for the 3'-5' proofreading exonuclease activity in *E. coli,* whereas it is inactive in *Thermus aquaticus* [22]. To elucidate whether CDA *polymerase I* has a proofreading potential, we modeled its fragment containing the putative proofreading domain and the full length polymerase. The closest structural analogs (Table S10C) of the ribonuclease H superfamily domain were detected in three DNA polymerases: i) *E. coli* [23], ii) apicoplasts of *Plasmodium falciparum* [24] and iii) *Mycolicibacterium smegmatis (PubMed: 32034423).* The corresponding percent sequence identities in the structurally aligned region were: 20.1, 21.8 and 22.2%, respectively. The polymerases from *E. coli* and *P. falciparum* possess 3'-5' proofreading activity, while the polymerase from *M. smegmatis* lacked it. The analysis of potential active site residues (Fig. S7) showed that even though this domain is structurally close to proofreading activity domains, it lacks

acidic catalytic residues, as is in the case of *M. smegmatis*. Structural analysis indicates that the CDA polymerase I is unlikely to possess 3'-5' proofreading activity.

***In vitro* analyses of DNA polymerases I, IV and X**

Cloning. Plasmid DNAs containing genes of polymerase I (Pol I), polymerase IV and polymerase X were synthesized by Invitrogen™ GeneArt®. Insert DNAs containing polymerase genes were amplified from plasmids using Invitrogen™ Platinum™ SuperFi™ Green DNA Polymerase (#10488-085). Amplified DNA fragments were extracted from agarose gel using Thermo Scientific™ GeneJET™ Gel Extraction and DNA Cleanup Micro Kit (#K0831). Ligation independent cloning of desired genes was performed using Thermo Scientific™ aLICator™ LIC Cloning Set1 (#K1271). Plasmid constructs were transferred into *E. coli* strain DH10B using calcium chloride transformation method [25]. Colony PCR was performed with Thermo Scientific™ DreamTaq™ Green DNA Polymerase (#EP0711) to screen for plasmids carrying the desired genes. Plasmid DNAs were isolated from overnight bacterial culture using Thermo Scientific™ GeneJET™ Plasmid Miniprep Kit (#K0502). Successful transformants were confirmed by Sanger sequencing of plasmid constructs.

Solubility. Plasmid constructs were transformed into *E. coli* protein expression strain ER2566. One mL of overnight bacterial cultures were transferred to separate 25 mL aliquots of LB medium with ampicillin (100 μg/mL) and incubated with shaking until each culture's optical densities (OD600) reached 0.5-0.8. The media was cooled down to 16°C, and protein expression was induced by adding IPTG to a final concentration of 2 mM and then incubating at 16°C with shaking overnight. Cells were collected by centrifugation at 20,000 xg for 15 min at 4°C and stored at -20°C. 0.3 g of each biomass were resuspended in 1 mL of lysis buffer (50 mM Tris-HCl pH 8,

300 mM NaCl, 2 mM EDTA, 0.5 % Elugent™, 1 mM DTT, 1X Halt™ Protease Inhibitor Cocktail) and sonicated (Vibra-Cell™ Ultrasonic Liquid Processor) on ice using pulse mode (9 seconds on, 9 seconds off) and 40% amplitude for 3 min. 100 µL of lysate was centrifuged at 22,000 ×g. The solubility of all three polymerases was determined by SDS-PAGE analysis of fresh cell lysate fraction, its supernatant (soluble fraction) taken after centrifugation as well as pellets (insoluble fraction) resuspended in 0.1 % SDS solution. Results showed that polymerase I was a soluble protein fraction. However, polymerases IV and X were insoluble, despite testing several different bacterial culture growth conditions, such as different media composition, temperature of protein expression induction, and different concentrations of IPTG. Furthermore, we tested protein co-expression with molecular chaperones, which might assist conformational folding of polymerases. Unfortunately, polymerase IV and X proteins remained insoluble. Therefore, polymerase I became the focus of further experiments.

Production of purified polymerase I. Plasmid constructs were transformed into *E. coli* protein expression strain ER2566. Eight milliliters of overnight bacterial cultures were transferred to separate aliquots of 500 mL of LB media with ampicillin (100 µg/mL) and incubated with shaking until each culture's optical densities (OD600) reached 0.5-0.8. The media was cooled down to 16°C, and protein expression was induced by adding IPTG to a final concentration of 2 mM and then incubating at 16°C with shaking overnight. Cells were collected by centrifugation at 20,000 ×g for 15 min at 4°C and stored at -20°C. Two grams of bacterial biomass were resuspended in 8.4 mL of buffer A (20 mM Tris-HCl pH 8, 50 mM NaCl, 2 mM EDTA, 0.5% Elugent™, 1 mM DTT) and 80 µL of 100× Halt™ Protease Inhibitor Cocktail. Cells were sonicated (Vibra-Cell™ Ultrasonic Liquid Processor) on ice using pulse mode (10 seconds on, 10 seconds off) and 50% amplitude for 7.5 minutes. Cell debris were pelleted by centrifugation at 30,000 ×g for 30

min at 4°C. Supernatant was passed through 0.2 μm filters and collected and stored at 4°C for protein purification step.

Polymerase I protein was purified from cleared cell lysate by chromatography using an ion-exchange POROS HQ 50 (Applied Biosystems resin) column as a first purification stage. Loaded cell lysate solution was eluted in 50 - 1000 mM NaCl gradient. Collected protein fractions were analyzed by SDS-PAGE. Selected fractions were combined, slowly mixed with equal volume of 3 M $(NH_4)_2SO_4$, loaded on hydrophobic POROS Ethyl column (Thermo Scientific resin) and eluted in a 1500-0 mM $(NH_4)_2SO_4$ gradient. Collected protein elution fractions were analyzed by SDS-PAGE. Selected fractions were combined, concentrated 13-fold by Amicon Ultra 15K concentrators and then dialyzed in storage buffer (20 mM Tris-HCl pH 8, 100 mM KCl, 0.1 mM EDTA, 0.5% Nonidet P40™, 0.5% Tween 20™, 50% glycerol) overnight at 4°C. After dialysis, protein sample was stored at -20°C.

Polymerase I fidelity evaluation. DNA substrate for polymerase I primer extension reaction was generated by transforming *E. coli* bacteria with M13 phage. A single infected bacterial colony was used for growing biomass. Extracellular circular single-stranded M13 DNA (ssDNA) substrate was purified from growth media. Double-stranded DNA (dsDNA) was extracted from harvested bacteria cells as a sequencing reference control. The 2-fold concentrated ssDNA substrate for primer elongation reaction was prepared by annealing the DNA oligonucleotide M13_XbaI containing XbaI restriction endonuclease recognition sequence (5'-GCCTGCAGGTCGACT**CTAGA**GGATCCCCGGGTACCGAGC-3') (XbaI recognition sequence is bolded) to the single-stranded M13mp18 DNA template. The mixture contained 2 μM primer M13_XbaI and 2 μg single stranded M13mp18 DNA in different 2X reaction buffers accordingly to each polymerase used in this experiment. Substrate for Taq DNA polymerase

primer extension reaction was hybridized in supplied Taq DNA Polymerase reaction buffer (Thermo Scientific, cat. no. B33), with $(NH_4)_2SO_4$, and substrate for Bst DNA Polymerase Full Length (NEB cat. no. M0328S) primer extension reaction. Finally, the substrate for CDA DNA polymerase I primer extension reaction was prepared in 2× buffer with concentrations of 150 mM Tris-HCl pH 8.8, 40 mM $(NH_4)_2SO_4$, 3.2 mM $MgCl_2$ and 0.02 % Tween 20™. All substrate hybridization mixtures were incubated at 99°C for 5 min and cooled down to room temperature. ssDNA substrate with annealed primer as well as the dsDNA control were linearized by digestion with 2 μL of FastDigest XbaI (Thermo Scientific, cat. no. FD0684) restriction enzyme for 60 min at 37°C.

50 μL of CDA DNA polymerase I primer elongation reaction mix (25 μL of corresponding hybridized substrate, 0.2 μM dNTP mix (Thermo Scientific™ cat. No. R0192) and 1.25 U of Polymerase I) was incubated at 60°C for 20 min. 50 μL of *Taq* DNA polymerase primer elongation reaction mix (25 μL of corresponding hybridized substrate, 0.2 μM dNTP mix and 1.25 U of *Taq* polymerase) was incubated at 72°C for 90 min. 50 μL of *Bst* DNA polymerase primer elongation reaction mix (25 μL of corresponding hybridized substrate, 0.25 μM dNTP mix, 4 U of *Bst* polymerase and nuclease-free water up to 50 μL) was incubated at 65°C for 15 min. The ~7 kb DNA products were purified with Thermo Scientific™ GeneJET™ PCR Purification Kit (cat. no. K0701) and treated with *RecJ$_f$* exonuclease (NEB cat. no. M0264S) in 1× NEBuffer 2 to hydrolyse 5‘ssDNA ends (10 U to 1 μg of DNA product) if the primer was not extended completely. After this step, the DNA product was again purified. The ~7kb fragment was then processed in a similar manner as described here (https://www.thermofisher.com/lt/en/home/life-science/pcr/pcr-enzymes-master-mixes/platinum-high-fidelity-pcr-enzyme.html) (R. Sukackaitė unpublished), with the main difference being that we only extended the DNA template once.

Polymerase I 3'-5' exonuclease activity evaluation. Experimental validation of 3'-5' proofreading activity was performed by hydrolysis reaction of phosphothiolated DNA-DNA oligoduplex (Metabion). The oligoduplex with 12 deoxyribonucleotide 5' overhang (hybridized SEQ ID NO: 1 and SEQ ID NO: 2) was comprised of 42 base oligonucleotide and 30 base 6-fluorescein amidite (FAM) labeled oligonucleotide, with 3 phosphodiester backbone linkages replaced by phosphothiolates (PTO) at the 5' end to block exonuclease activity.

SEQ ID NO: 1: 5'-GCGCGTCCCATTCGCCAATCGTTTTAGAGCTGTGTTGTTTCG-3'

SEQ ID NO: 2: 5'-CGAAACAACACAGCTCTAAAACGATTGGCG-3'

Reactions were performed in a 30 μL reaction volume containing 75 mM Tris-HCl pH 8.8 at 25°C, 20 mM $(NH_4)_2SO_4$, 0.01% (v/v) Tween 20™, 0.2 mM dNTP, 1.6 mM $MgCl_2$, and 50 nM DNA-DNA oligoduplex and incubated at 60°C for 30 min using 15 U of Polymerase I. Analogous reactions were performed using SuperFi™ and *Taq* DNA polymerases. Reactions were quenched by adding 10 μL of reaction mixture to 14 μL STOP solution (10 mM EDTA, 98% (v/v) Formamide, 10 mg/ml Blue Dextran) and cooling the reaction down on ice. Then 10 μL of the samples were analyzed by denaturing urea (7 M) polyacrylamide gel (15%) electrophoresis at 50°C. Phi29 was not included in these experiments because of insufficient fidelity sensitivity of the method applied.

Phylogeny of DNA polymerase I

DNA polymerase I orthologs were identified using OrthoDB v10 [26]. Initially all orthologs of *Firmicutes* phylum were selected. Only proteins having a description "DNA polymerase I" were chosen, and 200 were randomly selected for further phylogenetic analyses.

The protein set was supplemented by the sequence of DNA polymerase I from *Ca.* D. audaxviator and two orthologous sequences from Deinococcus–Thermus phylum that were used as an out-group (DNA polymerases I from T*hermus aquaticus* Y51MC23 and *Deinococcus geothermalis* DSM 11300). Sequence alignment was produced using PSI-Coffee [27]. The maximum likelihood tree was calculated by raxml-ng v. 0.9.0 [28], using command line option "LG+G8+F" for model selection. A total of 36 searches were conducted: 18 using maximum parsimony starting trees, and 18 using random starting trees. The best tree based on the maximum likelihood score was chosen for visualization and analysis.

Supplemental Table S1. Properties of CDA SAG assemblies.

SAG microplate	Raw read count	Contig count	Assembly size, bp	GC content, %	estimated completeness, %	Estimated genome size, bp
AC-311-A22	10,382,790	99	752,467	59.8	20.06	3,751,081
AC-311-B02	8,055,884	80	576,279	60.4	32.52	1,772,075
AC-311-B17	11,304,695	66	354,904	60.8	0.00	NA
AC-311-B19	27,959,109	46	350,903	60.0	17.24	2,035,400
AC-311-C23	9,996,848	62	353,875	60.6	8.33	4,248,199
AC-311-D21	10,967,337	74	635,258	59.2	23.31	2,725,259
AC-311-E17	28,940,604	50	419,342	59.9	23.80	1,761,941
AC-311-F23	8,087,508	26	307,609	60.0	10.34	2,974,941
AC-311-G03	7,908,440	69	558,345	59.9	14.50	3,850,655
AC-311-G10	9,306,999	123	1,514,250	60.8	63.94	2,368,235
AC-311-I15	9,228,121	97	785,841	59.8	17.32	4,537,188
AC-311-J18	9,449,541	109	1,113,432	60.0	42.52	2,618,607
AC-311-K13	39,232,262	91	645,965	60.2	29.31	2,203,906
AC-311-L03	29,573,500	99	1,339,388	60.5	66.61	2,010,791
AC-311-N04	11,151,563	93	693,860	59.3	21.58	3,215,291
AC-311-N21	10,306,584	76	472,769	60.2	8.31	5,689,157
AG-494-A02	10,243,307	30	129,527	58.8	1.72	7,530,639
AG-494-A04	8,551,747	71	584,322	61.0	16.40	3,562,939
AG-494-A08	8,381,173	54	322,750	59.7	8.95	3,606,145
AG-494-A16	7,355,847	57	361,180	59.7	18.97	1,903,953
AG-494-A22	7,524,484	85	706,110	60.7	30.97	2,279,980
AG-494-A23	8,935,783	57	450,624	60.3	20.33	2,216,546
AG-494-B04	8,573,963	56	466,776	60.2	18.97	2,460,600
AG-494-B19	6,453,662	69	398,831	60.0	16.38	2,434,865
AG-494-B22	4,973,632	66	593,615	60.2	27.71	2,142,241
AG-494-C02	6,094,245	68	366,154	59.3	12.07	3,033,587
AG-494-D02	4,840,266	69	548,527	60.5	18.51	2,963,408
AG-494-D10	5,407,148	72	517,559	59.9	20.06	2,580,054
AG-494-D15	4,397,456	71	454,453	60.5	19.62	2,316,274
AG-494-D17	3,511,547	77	793,300	60.5	18.65	4,253,619
AG-494-E03	12,184,901	60	479,840	59.7	16.05	2,989,657
AG-494-E06	7,248,570	95	561,382	60.0	26.35	2,130,481
AG-494-E09	10,252,791	65	485,587	60.2	13.32	3,645,548
AG-494-E23	6,989,456	54	327,393	60.0	16.14	2,028,457
AG-494-F03	8,002,230	71	425,626	60.1	20.67	2,059,148
AG-494-F05	7,407,575	35	135,612	59.0	3.45	3,930,782
AG-494-F06	7,087,626	41	240,870	60.7	0.00	NA
AG-494-F10	6,259,884	28	138,623	59.2	8.62	1,608,155
AG-494-F14	6,129,647	60	410,884	60.0	0.00	NA
AG-494-G09	8,374,765	81	519,271	59.7	20.97	2,476,256
AG-494-G11	7,083,011	22	160,364	57.7	0.00	NA
AG-494-G13	6,740,998	78	579,432	59.8	23.35	2,481,507
AG-494-G16	4,882,669	68	426,879	60.6	18.97	2,250,284
AG-494-G22	4,218,573	41	187,551	60.7	10.34	1,813,839
AG-494-I05	6,485,253	71	656,422	60.1	36.14	1,816,330
AG-494-I07	7,468,423	50	322,706	60.2	0.00	NA
AG-494-I13	5,651,321	24	183,856	60.1	4.83	3,806,542
AG-494-I21	4,651,695	51	282,876	59.9	6.94	4,076,023
AG-494-I22	4,863,470	69	600,211	59.7	29.41	2,040,839
AG-494-I23	4,762,666	38	156,406	58.1	8.62	1,814,454
AG-494-J02	4,264,963	77	673,012	60.0	17.24	3,903,781
AG-494-J07	4,011,803	83	707,405	60.4	29.41	2,405,321
AG-494-J09	3,755,369	57	345,515	60.1	16.56	2,086,443
AG-494-J17	3,645,478	56	353,793	60.1	16.38	2,159,908
AG-494-J18	3,400,874	64	556,829	59.9	27.38	2,033,707

AG-494-J23	4,017,725	27	228,903	60.6	8.62	2,655,487
AG-494-K08	4,810,933	60	448,838	59.7	13.79	3,254,807
AG-494-K15	5,999,471	58	504,867	60.3	25.86	1,952,308
AG-494-K16	3,997,090	58	326,978	60.1	8.71	3,754,052
AG-494-K20	4,963,258	51	413,933	60.4	16.61	2,492,071
AG-494-L11	4,000,687	67	523,250	60.3	21.94	2,384,913.
AG-494-L18	3,943,186	68	619,703	60.6	32.76	1,891,645
AG-494-L22	4,648,073	67	438,996	59.6	12.65	3,470,324
AG-494-M04	5,752,612	92	881,314	60.6	45.73	1,927,211
AG-494-M15	6,290,588	26	127,598	57.8	<1	NA
AG-494-M17	6,041,051	22	115,255	58.8	0.00	NA
AG-494-N17	4,150,012	67	484,562	60.0	24.68	1,963,379
AG-494-N19	4,244,717	33	234,939	60.0	0.94	24,993,510
AG-494-N20	2,597,663	65	729,890	61.1	28.58	2,553,848
AG-494-N21	4,518,587	73	675,969	60.4	22.91	2,950,541
AG-494-N22	4,745,371	80	549,262	60.3	27.59	1,990,801
AG-494-O11	5,891,986	70	532,305	60.1	27.59	1,929,340
AG-494-O14	4,426,597	67	392,652	59.9	11.21	3,502,694
AG-494-O17	6,359,939	34	153,495	60.0	3.45	4,449,130
AG-494-P02	11,356,941	36	193,849	59.4	8.10	2,393,197
AG-494-P06	3,994,767	28	150,224	59.9	7.90	1,901,569
AG-494-P09	3,845,969	79	662,045	60.4	38.62	1,714,254
AG-494-P13	3,791,614	70	508,033	60.2	31.50	1,612,803
AG-494-P16	8,382,287	85	898,486	60.6	40.50	2,218,483
AG-494-P23	3,970,780	52	411,604	59.6	17.99	2,287,959
AG-502-B21	23,206,864	101	749,765	60.6	29.31	2,558,051
AG-502-D20	34,397,922	31	244,289	60.9	12.07	2,023,935
AG-516-A17	7,068,729	37	281,718	60.1	24.14	1,167,017
AG-516-B22	7,074,829	44	253,028	60.3	12.07	2,096,338
AG-516-E23	5,978,728	34	149,580	59.7	0.00	NA
AG-516-K20	4,739,535	27	172,019	60.4	0.00	NA
AG-516-N05	4,226,015	38	276,924	59.9	10.83	2,557,008
AG-516-O06	4,534,405	36	321,358	60.6	10.34	3,107,911
AG-516-O16	4,060,461	51	376,971	59.8	22.10	1,705,751
AG-516-O18	14,238,265	65	385,562	60.7	16.14	2,388,859
AG-516-P11	5,506,787	52	407,475	59.0	16.06	2,537,204
AG-516-P13	12,263,738	36	264,172	59.8	5.17	5,109,709
AG-592-C13	13,678,693	64	1,022,345	60.6	27.24	3,753,102
AG-592-F10	12,623,992	54	357,379	59.2	0.00	NA
AG-592-F16	16,155,790	50	623,893	60.0	18.97	3,288,840
AG-592-G18	12,761,837	3	109,045	61.8	8.33	1,309,063
AG-592-J19	13,064,167	93	998,863	60.1	43.10	2,317,547
AH-151-A17	10,195,597	34	584,946	61.7	29.31	1,995,721
AH-151-B20	8,926,977	118	1,301,876	60.3	48.54	2,682,068
AH-151-C22	8,322,096	40	164,820	62.1	3.45	4,777,391
AH-151-E10	6,333,723	52	358,395	60.7	6.90	5,194,130
AH-151-E15	7,060,124	90	775,402	60.0	32.72	2,369,810
AH-151-F07	6,193,907	24	131,304	59.9	6.03	2,177,512
AH-151-I21	9,167,343	27	152,695	62.3	15.52	983,859.54
AH-151-J06	7,533,598	32	157,198	56.9	3.99	3,939,799
AH-151-K02	5,938,552	46	687,636	60.6	32.53	2,113,851
AH-151-N03	6,285,424	60	483,139	59.4	25.86	1,868,286
AH-151-O13	4,992,778	52	270,904	60.1	0.00	NA
AH-151-P19	8,618,713	64	598,910	60.7	17.24	3,473,955
AH-147-A10	5,418,224	28	171,161	60.0	6.37	2,686,985
AH-147-B11	6,506,607	42	203,729	58.6	6.90	2,952,594
AH-147-B13	4,326,890	31	182,323	60.4	0.00	NA
AH-147-B14	6,134,505	42	212,629	61.0	4.17	5,099,016
AH-147-C08	5,160,549	24	126,188	60.3	7.63	1,653,840

AH-147-C15	34,022,815	33	214,111	60.8	13.79	1,552,654
AH-147-F15	4,470,487	32	220,864	59.6	10.34	2,136,015
AH-147-J03	3,733,757	22	162,488	61.2	10.34	1,571,450
AH-147-K13	5,558,236	20	137,076	60.1	0.00	NA
AH-147-K20	3,931,213	49	217,793	58.6	0.63	34,570,317
AH-147-L04	4,339,666	66	418,440	60.0	16.34	2,560,832
AH-147-N07	4,628,580	71	472,765	60.0	10.34	4,572,195
AH-147-O15	4,835,689	21	102,260	60.1	0.00	NA
AH-147-P10	4,337,790	55	302,746	59.8	8.62	3,512,134
AG-720-E19	959,905	120	959,905	60.5	55.17	1,739,903
AG-720-I18	568,678	61	568,678	60.7	26.72	2,128,285
AG-720-J22	1,505,580	105	1,505,580	60.8	54.57	2,758,988

Supplemental Table S2. Geochemistry of field samples.

Sample Name	Inyo-BLM 1	Beatrix	Tau Tona	Mponeng	BY-1R
**SAG number**	**AG-147/AG-151**	**AG-516**	**AC-311/AG-592**	**AG-494/AG-502**	**AG-720**
**Latitude**	36.400	-28.238	-26.417	-26.438	58.450
**Longitude**	-116.469	26.797	27.427	27.431	85.028
**Depth**	750 mbls	1339 mbls	3136 mbls	3402 mbls	2005 mbls
**T (°C)**	57.2	36.9	49.2	65	40.2-44.8
**pH**	6.92	8.8	8	8.24	7.92-8.25
**Eh (mV)**	-242	-97.6	-337.3	-89.2	-279
**TDS (ppt)**	NA	4.47	0.30	1.3	NA
**DOC (M)**	3.59E-05	1.6E-05	3.9E-05	NA	NA
**DIC (M)**	4.37e-3	5.1e-4	7.46e-4	NA	NA
**Formate (M)**	9.12E-07	8.40E-06	9.95E-07	6.20E-04	NA
**Acetate (M)**	1.67E-06	1.63E-06	1.07E-07	1.12E-03	NA
**Lactate (M)**	NA	<1.1e-6	<1.1e-6	<5.60E-04	NA
**Propanoate (M)**	1.07E-05	<1.37e-6	<1.37e-6	<3.80E-04	NA
**F (M)**	2.09E-04	9.05E-05	9.19E-05	5.95-05	6.1E-04
**Cl (M)**	1.48E-03	6.98E-02	3.22E-03	2.14E-02	24.5E-03
**Br (M)**	1.2E-06	1.66E-04	7.35E-06	4.0E-05	3.88E-05
**SO_4^{2-} (M)**	1.64E-03	1.37E-04	1.29E-04	<5.2E-05	<5.2E-05
**$S_2O_3^{2-}$ (M)**	NA	4.91E-05	NA	NA	NA
**HS^- (M)**	<2.93E-05	1.47E-05	2.87E-07	NA	NA
**Total S M ICP (M)**	NA	4.9E-04	1.09E-03	NA	NA
**PO_4^{3-} (M)**	4.21E-07	<1.05e-6	1.58E-07	<1.04E-07	NA
**Total P M ICP (M)**	NA	1.82E-04	<3.13E-07.	NA	NA
**NO_{2-} (M)**	<2.17E-07	3.91E-06	<2.2e-6	<2.0E-05	NA
**NO_{3-} (M)**	<3.23E-07	3.71E-07	1.03E-07	<8.0E-05	<4.84E-05
**NH_4^+ (M)**	1.22E-05	8.32E-05	<5.6E-07	NA	NA
**Na^+ (M)**	9.66E-03	7.80E-02	3.09E-03	NA	28.75E-03
**K^+ (M)**	4.35E-04	7.29E-04	4.02E-05	NA	7.72E-05
**Mg^{2+} (M)**	4.98E-04	5.60E-05	4.44E-05	NA	5.76E-06
**Ca^{2+} (M)**	7.91E-04	2.87E-03	5.09E-04	NA	0.24 E-02
**Sr^{2+} (M)**	NA	7.99E-06	2.85E-06	NA	11.6E-06
**Ba^{2+} (M)**	7.98E-07	9.67E-06	4.73E-07	NA	1.63E-06
**Al^{3+} (M)**	2.96E-07	1.67E-06	1.37E-06	NA	NA
**Si^{4+} (M)**	1.56E-03	3.97E-04	4.39E-04	NA	4.54E-04
**Mn (M)**	1.74E-06	6.55E-07	<1.0E-08	NA	1.71E-07
**Fe (M)**	1.43E-06	2.51E-07	2.51E-07	NA	2.13E-06
**Mo (M)**	5.56E-07	2.08E-08	<1.04E-08	NA	NA
**Cr (M)**	NA	3.85E-08	<1.9E-08	NA	NA
**Co (M)**	5.71E-09	5.09E-08	<8.5E-09	NA	NA
**Ni (M)**	1.57E-07	1.02E-06	1.12E-06	NA	NA
**Cu (M)**	9.44E-09	5.7E-08	<1.6E-08	NA	NA

**Zn (M)**	6.12E-08	3.06E-08	7.65E-08	NA	NA
**W (M)**	NA	2.5E-07	NA	NA	NA
**As (M)**	1.04E-06	3.87E-07	<1.3E-08	NA	NA
**U (M)**	8.4E-10	1.26E-08	<4.2E-09	NA	NA
**He (M)**	NA	5.07E-05	3.78E-05	NA	NA
**H_2 (M)**	3.00E-06	1.30E-07	4.32E-08	NA	NA
**O_2 (M)**	<6.25E-06	<3.1e-7	2.09E-05	NA	6.90%
**N_2 (M or %)**	3.60E-03	3.56E-04	3.34E-04	NA	32%
**CO (M or %)**	6.00E-07	1.80E-07	9.83E-08	NA	0.20%
**CH_4 (M or %)**	1.30E-05	1.95E-03	9.47E-04	NA	60.20%
**C_2H_6 (M)**	NA	1.64E-06	NA	NA	NA
**C_3H_8 (M)**	NA	1.74E-07	NA	NA	NA
**i-C_4H_{10} (M)**	NA	<1.19e-7	NA	NA	NA
**n-C_4H_{10} (M)**	NA	<1.49e-7	NA	NA	NA
**i-C_5H_{12} (M)**	NA	<6.8e-8	NA	NA	NA
**n-C_5H_{12} (M)**	NA	<6.8e-8	NA	NA	NA
**CO_2 (M)**	NA	NA	1.04E-05	NA	NA
**NMHC (M)**	NA	NA	1.62E-06	NA	NA
**N_2O (M)**	NA	NA	NA	NA	NA

Supplemental Table S3. Individual *D. audaxviator* SAG assembly alignments to MP104C genome.

SAG	ANI	% aligned to MP104C	SAG	ANI	% aligned to MP104C
AG-592-G18	100.00	100.00	AG-494-E06	99.83	95.05
AH-151-A17	99.99	99.71	AG-494-M04	99.79	94.97
AC-311-L03	99.99	99.58	AG-494-K15	99.82	94.96
AG-494-K20	99.94	99.57	AG-494-E09	99.90	94.89
AC-311-G10	99.98	99.53	AG-494-I22	99.80	94.87
AH-147-J03	99.99	99.44	AG-516-E22	99.66	94.79
AG-592-F16	99.99	99.41	AG-494-E03	99.85	94.74
AH-151-N03	99.99	99.35	AG-494-F14	99.80	94.73
AH-147-K13	99.99	99.33	AG-494-J09	99.79	94.70
AG-592-F10	100.00	99.16	AG-494-P13	99.88	94.64
AC-311-E17	99.99	99.04	AG-494-M17	99.99	94.56
AH-147-A10	100.00	99.04	AC-311-G03	99.99	94.20
AH-147-C15	99.99	99.02	AG-494-O14	99.85	94.16
AC-311-N04	99.99	98.98	AG-494-G22	99.92	94.02
AG-516-A17	99.99	98.87	AG-494-N17	99.94	93.78
AG-720-E19	99.95	98.80	AG-494-J07	99.80	93.74
AG-592-J19	99.98	98.78	AG-494-J02	99.84	93.67
AC-311-I15	99.98	98.71	AH-151-I21	99.75	93.62
AG-494-J17	99.83	98.68	AG-494-C02	99.86	93.56
AG-494-L18	99.76	98.68	AH-151-K02	99.91	93.35
AH-151-P19	99.99	98.67	AG-494-P23	99.84	93.30
AC-311-B19	99.99	98.64	AG-494-P02	99.97	93.07
AC-311-D21	99.99	98.45	AG-494-I05	99.86	92.98
AG-494-J23	99.83	98.44	AG-494-F10	99.91	92.91
AC-311-N21	99.99	98.32	AG-494-J18	99.82	92.71
AG-494-D17	99.88	97.85	AG-494-G16	99.87	92.65
AG-502-B21	99.88	97.78	AH-147-B13	99.99	92.63
AG-494-D10	99.94	97.71	AH-147-O15	99.48	92.51
AG-494-K16	99.91	97.67	AG-516-N05	99.53	92.42
AG-494-N20	99.93	97.62	AG-494-A16	99.82	92.39
AG-502-D20	99.83	97.60	AG-494-G13	99.88	92.31
AG-494-B22	99.93	97.57	AG-494-N22	99.89	92.29
AG-494-N21	99.78	97.51	AG-494-K08	99.85	92.24
AH-147-B11	99.97	97.12	AG-494-F03	99.84	92.10
AC-311-F23	100.00	97.04	AG-494-A02	99.92	92.01
AH-147-C08	99.54	96.96	AG-516-O16	99.73	91.91
AC-311-B02	99.98	96.87	AH-147-L04	99.99	91.75
AG-494-A22	99.87	96.86	AH-151-O13	99.92	91.73
AC-311-C23	99.95	96.85	AG-494-L22	99.92	91.61

AC-311-B17	99.99	96.76	AH-147-P10	99.99	91.47
AG-720-J22	99.94	96.76	AG-494-G09	99.78	91.41
AG-494-I13	99.94	96.67	AH-147-B14	99.99	91.20
AG-720-I18	99.84	96.65	AG-494-F06	99.65	91.08
AH-147-F15	99.99	96.57	AG-494-I07	99.77	90.22
AG-494-P16	99.83	96.55	AG-516-O18	99.63	89.98
AC-311-K13	99.97	96.52	AG-516-B22	99.74	89.88
AH-147-N07	99.98	96.33	AG-494-E23	99.83	89.68
AC-311-A22	99.99	96.14	AG-494-F05	99.88	89.64
AG-494-D02	99.73	96.05	AG-494-M15	99.78	89.62
AG-494-D15	99.92	95.96	AG-494-G11	99.45	89.60
AG-494-O17	99.74	95.78	AG-516-O06	99.52	89.55
AC-311-J18	99.99	95.74	AH-151-B20	99.95	89.52
AG-494-P09	99.81	95.63	AG-516-P11	99.63	89.47
AG-494-O11	99.87	95.43	AH-151-F07	99.99	88.89
AG-494-B19	99.90	95.37	AH-151-J06	99.98	88.79
AH-151-E15	99.92	95.27	AG-494-P06	99.66	88.14
AG-592-C13	99.99	95.23	AG-516-P13	99.69	87.67
AG-494-B04	99.89	95.15	AG-494-I23	99.97	86.60
AG-494-A23	99.91	95.13	AH-151-C22	99.97	85.47
AG-494-A08	99.93	95.11	AG-494-I21	99.90	85.26
AG-494-L11	99.73	95.11	AH-151-E10	99.97	82.13
AG-494-N19	99.87	95.11	AH-147-K20	99.99	74.60
AG-494-A04	99.76	95.08	AG-516-K20	99.20	73.07

Supplemental Table S4. Statistics for within-site and between-site SAG ANI comparisons used to calculate F_{ST}.

**Tau Tona**		**Mponeng**		**Beatrix**		**Inyo-BLM 1**		**BY-1R**	
Comparisons	206	Comparisons	2089	Comparisons	40	Comparisons	249	Comparisons	3
Average ANI	99.96	Average ANI	99.97	Average ANI	99.64	Average ANI	99.96	Average ANI	99.98
Min	99.61	Min	99.22	Min	98.24	Min	98.43	Min	99.96
Max	100	Max	100	Max	99.99	Max	100	Max	99.99
STD	0.05	STD	0.04	STD	0.32	STD	0.12	STD	0.01
Error	0.003	Error	0.001	Error	0.05	Error	0.01	Error	0.01

**Tau Tona <> Mponeng**		**Beatrix <> Mponeng**		**Inyo-BLM 1 <>Beatrix**		**Inyo-BLM 1 <> BY-1R**		**Tau Tona <>Beatrix**	
Comparisons	1349	Comparisons	617	Comparisons	203	Comparisons	78	Comparisons	203
Average ANI	99.79	Average ANI	99.56	Average ANI	99.67	Average ANI	99.92	Average ANI	99.63
Min	97.63	Min	96.90	Min	97.75	Min	99.58	Min	98.05
Max	100	Max	100	Max	100	Max	100	Max	100
STD	0.22	STD	0.37	STD	0.35	STD	0.09	STD	0.32
Error	0.01	Error	0.01	Error	0.02	Error	0.01	Error	0.02
P(T<=t) two-tail	0.01	P(T<=t) two-tail	0.00	P(T<=t) two-tail	0.00	P(T<=t) two-tail	0.08	P(T<=t) two-tail	0.00
F	1.24	F	0.02	F	7.84	F	56.36	F	0.02
P(F<=f) one-tail	0.02	P(F<=f) one-tail	0.00	P(F<=f) one-tail	0.00	P(F<=f) one-tail	0.018	P(F<=f) one-tail	0.00

**Inyo-BLM 1 <> Mponeng**		**BY-1R <> Beatrix**		**Tau Tona <> Inyo-BLM**		**BY-1R <> Mponeng**		**Tau Tona <> BY-1R**	
Comparisons	1506	Comparisons	30	Comparisons	515	Comparisons	198	Comparisons	63
Average ANI	99.76	Average ANI	99.61	Average ANI	99.86	Average ANI	99.76	Average ANI	99.86
Min	96.66	Min	98.63	Min	97.93	Min	99.08	Min	99.05
Max	100	Max	99.98	Max	100	Max	100	Max	100
STD	0.34	STD	0.30	STD	0.28	STD	0.15	STD	0.14
Error	0.01	Error	0.05	Error	0.01	Error	0.01	Error	0.02
P(T<=t) two-tail	0.01	P(T<=t) two-tail	0.00	P(T<=t) two-tail	0.01	P(T<=t) two-tail	0.61	P(T<=t) two-tail	0.61
F	0.15	F	441.75	F	0.18	F	8.42	F	10.41
P(F<=f) one-tail	0.00	P(F<=f) one-tail	0.00	P(F<=f) one-tail	0.00	P(F<=f) one-tail	0.11	P(F<=f) one-tail	0.09

Supplemental Table S5. Proteins that are unique for individual field samples.

Site	Annotation and KO number
Beatrix	agrB; accessory gene regulator B
Beatrix	gmd; GDPmannose 4,6-dehydratase [EC:4.2.1.47]
Beatrix	transposase
Inyo-BLM 1	cpaF; pilus assembly protein CpaF
Inyo-BLM 1	parB; chromosome partitioning protein, ParB family
Inyo-BLM 1	parM; plasmid segregation protein ParM
Inyo-BLM 1	putative transposase
Inyo-BLM 1	smpB; SsrA-binding protein
Mponeng	ABC-2.P; ABC-2 type transport system permease protein
Mponeng	ACR3; arsenite transporter
Mponeng	mazF; mRNA interferase MazF [EC:3.1.-.-]
Mponeng	phnD; phosphonate transport system substrate-binding protein
Mponeng	phnE; phosphonate transport system permease protein
Mponeng	phnG
Mponeng	phnH
Mponeng	phnI; alpha-D-ribose 1-methylphosphonate 5-triphosphate synthase subunit PhnI [EC:2.7.8.37]
Mponeng	phnJ; alpha-D-ribose 1-methylphosphonate 5-phosphate C-P lyase [EC:4.7.1.1]
Mponeng	phnK
Mponeng	phnL; alpha-D-ribose 1-methylphosphonate 5-triphosphate synthase subunit PhnL [EC:2.7.8.37]
Mponeng	phnM; alpha-D-ribose 1-methylphosphonate 5-triphosphate diphosphatase [EC:3.6.1.63]
Mponeng	relE; mRNA interferase RelE/StbE

Supplemental Table S6. Putative viral contigs identified in CDA SAGs.

SAG	Site	Contig number	Contig length	Gene count	Viral gene count	Viral assembly number*
AC-311-A22	Tau Tona	16	12865	17	4	-
AC-311-D15	Tau Tona	19	8399	10	4	4
AC-311-J18	Tau Tona	9	25584	35	16	-
AG-494-A02	Mponeng	8	4943	4	2	3
AG-494-A04	Mponeng	39	2596	4	2	3
AG-494-A22	Mponeng	32	7397	9	3	3
AG-494-C02	Mponeng	30	4881	5	2	3
AG-494-L22	Mponeng	54	3129	5	2	5
AG-494-N17	Mponeng	57	2454	3	1	3
AG-494-N22	Mponeng	64	3192	5	2	5
AG-494-O17	Mponeng	16	4908	4	2	3
AG-502-B21	Mponeng	86	2842	5	2	5
AG-516-K20	Beatrix	8	7761	9	3	4
AG-720-I18	BY-1R	27	7178	14	2	1
AG-720-J22	BY-1R	35	15923	25	6	1
AH-147-K20	Inyo-BLM 1	14	5514	10	2	1
AH-147-N07	Inyo-BLM 1	22	8002	18	2	1
AH-147-O15	Inyo-BLM 1	14	3185	6	2	1
AH-151-C22	Inyo-BLM 1	4	6480	5	2	2
AH-151-E15	Inyo-BLM 1	61	4204	4	3	1
AH-151-E15	Inyo-BLM 1	10	19348	24	12	2
AH-151-F07	Inyo-BLM 1	3	9039	15	3	1
AH-151-J06	Inyo-BLM 1	6	7241	11	4	2
AH-151-O13	Inyo-BLM 1	30	3984	3	3	1
AH-151-O13	Inyo-BLM 1	8	8820	9	2	2

*Assembly number corresponds to Figure 3 in the main text.

Supplemental Table S7. Commonly used 16S rRNA primers and their binding efficiency to *D. audaxviator*.

Probe name	Sequence (5'-3')	Hybrid T°C	ΔG (kcal/mole)	H.E.
B27F	AGAGTTTGATCCTGGCTCAG	50	-11.9	0.9642
B1492R	GGTTACCTTGTTACGACTT	50	-10.8	0.8320
V4-7 - 530F	GTGCCAGCAGCAGCGG	56	-13.0	0.9931
V4-7 - 1073R	CTATGCGCCTTGCCAGCCCGCTCAG	56	-7.2	0.0144
V6 – 967F	CAACGCGAAGAACCTTACC	55	-11.9	0.9510
V6 – 1064R	CGACRRCCATGCANCACCT	55	-12.8	0.9886
Bact338	GCTGCCTCCCGTAGGAGT	48	-17.7	1.0000
V4-V5 - 518F	CCAGCAGCYGCGGTAAN	50	-15.4	0.9998
V4-V5 - 926R	CCGTCAATTCNTTTRAGT	50	-14.0	0.9985
V3-V5 - 341F	TCTACGGAAGGCTGCAG-7	mismatches		

Supplemental Table S8. Samples in the IMNGS database that contained sequence reads with ≥99% nucleotide similarity to the CDA 16S rRNA gene.

Sample ID	Sample Size	Description	Reads ≥99%	Reads ≥97%	Site description	Depth (m)	Age	Ref.
SRR1296092	321739	groundwater	792	836	anthropogenic CO_2 injection in basaltic aquifer, Iceland	400-500	0-400 kyr	[33-34]
SRR1686681	14018	groundwater	677	677	Paleozoic dolomite aquifer NNSS, Nevada, USA	973	Paleozoic	[35-38]
SRR1686680	22320	groundwater	160	160	Paleozoic dolomite aquifer Inyo-BLM 1, Nevada, USA	750-884	Paleozoic	[10, 35]
SRR1686678	27090	groundwater	94	94	Tertiary tuff and rhyolitic lava, NNSS, U12N 10 Tunnel, Nevada, USA	610-900	2.6-23 myr	[35, 39-40]
MW686394-MW686475	82	groundwater	1	1	ER-EC-11, Miocene tuff and rhyolitic lava, NNSS, Nevada, USA	1,264.3	2.6-23 myr	[41]
MW686477-MW686547	92	groundwater	0	2	ER-EC-12, Miocene tuff and rhyolitic lava, NNSS, Nevada, USA	1,240.2	2.6-23 myr	[42-43]
MW652324-MW652388	79	groundwater	0	2	ER-EC-13, Miocene tuff and rhyolitic lava, NNSS, Nevada, USA	991.4	2.6-23 myr	[43-44]
DRR054745	80256	groundwater	194	194	anaerobic sedimentary aquifer in Southeastern Kyushu, Japan, MR4	774-1054	Neogene-Paleogene	[45]
DRR054747	57043	groundwater	11	12	anaerobic sedimentary aquifer in Southeastern Kyushu, Japan, OYD	947-1240	Neogene-Paleogene	[45]
DRR054749	55702	groundwater	1	1	anaerobic sedimentary aquifer in Southeastern Kyushu, Japan, KG5	925-1047	Neogene-Paleogene	[45]
DRR075969	70174	groundwater	1	1	anaerobic sedimentary aquifer in Southeastern Kyushu, Japan, KGO	581-779	Neogene-Paleogene	[45]
DRR076033	66755	groundwater	9	9	deep aquifer of accretionary prism in Okinawa Island, Japan, LSH	800	Cretaceous	[46]
SRR097661	7306	hydrocarbon	1	1	coal bed produced water ConocoPhillips CG-1, Alberta	300-500	Upper Cretaceous	[47]
SRR097712	5906	hydrocarbon	1	1	coal bed produced water ConocoPhillips CG-1, Alberta	300-500	Upper Cretaceous	[47]
SRR2136237	38661	marine	2	2	marine methane seep, Hydrate Ridge, Oregon	0-0.15	modern	[48]
SRR2136222	42112	marine	1	1	marine methane seep, Hydrate Ridge, Oregon	0-0.15	modern	[48]
SRR2136223	41252	marine	1	1	marine methane seep, Hydrate Ridge, Oregon	0-0.15	modern	[48]
SRR2136239	41133	marine	1	1	marine methane seep, Hydrate Ridge, Oregon	0-0.15	modern	[48]
SRR2136246	51841	marine	1	1	marine methane seep, Hydrate Ridge, Oregon	0-0.15	modern	[48]
SRR6342056	123353	sediment	1	1	Guaymas Basin subsurface sediment (near hydrothermal vents)	3.75-3.8	modern	[49]

Supplemental Table S9. CDA mRNA sequences identified in the Beatrix metatranscriptome.

Data extracted from Lau et al. [29].

GenBank accession number	Description
WP_012302116.1	hypothetical protein
WP_012302142.1	polysulfide reductase
WP_041570778.1	hypothetical protein
WP_012301760.1	phosphate acyltransferase
WP_012302867.1	chemotaxis protein CheA
WP_012301254.1	heterodisulfide reductase
WP_012303013.1	pyruvate, water dikinase
WP_012302954.1	adenylylsulfate reductase subunit alpha
WP_012301582.1	hypothetical protein
WP_012303013.1	pyruvate, water dikinase
WP_012303262.1	sulfite reductase
WP_012301261.1	oxidoreductase
WP_012302046.1	ribonuclease J
WP_012301886.1	hypothetical protein
WP_012301255.1	hypothetical protein
WP_012303251.1	cobyrinic acid a,c-diamide synthase
WP_012302734.1	oxidoreductase
WP_012302864.1	FlgN family protein
WP_012302166.1	glutamate 2,3-aminomutase
WP_012301339.1	DNA repair protein RadA
WP_012302835.1	flagellar hook-length control protein
WP_012302866.1	chemotaxis protein CheB
WP_041570760.1	hypothetical protein
WP_012301343.1	bifunctional enzyme IspD/IspF
WP_012301342.1	twitching motility protein PilT
WP_012303041.1	hypothetical protein
WP_012303183.1	copper amine oxidase
ACA59173.1	3-oxoacyl-(acyl-carrier-protein) synthase III
ACA58862.1	protein of unknown function DUF199
ACA59293.1	diguanylate cyclase with PAS/PAC sensor
ACA59411.1	hypothetical protein Daud_0898
ACA59554.1	nickel-dependent hydrogenase, large subunit
ACA60530.1	4Fe-4S ferredoxin, iron-sulfur binding domain protein
ACA60277.1	flagellin domain protein

Supplemental Table S10A. Protein IDs for DNA polymerases and their active domains identified in CDA SAGs and other polymerases, along with annotated DNA repair mechanisms identified in CDA.

Protein (and domain)	InterProScan IDs	UniProt IDs	HHPRED PDB ID
DNA polymerase I - *Escherichia coli*		P00582	2KFZ
DNA polymerase I - *Thermus aquaticus*		P19821	1BGX
Apicoplast DNA Polymerase I - *Plasmodium falciparum*		Q8ILY1	5DKT
DNA polymerase I - *Geobacillus stearothermophilu*		E1C9K5	4YFU
DNA polymerase I '5-3' exonuclease domain	IPR002421		
DNA polymerase I Ribonuclease H superfamily domain	IPR036397		
DNA-directed DNA polymerase I, family A, palm domain	IPR001098		
DNA binding HU-beta (*hupB*)	IPR020816		
Uracil-DNA glycosylase	IPR002043		
MutL-MutS system	IPR032642-<br>IPR016151		
UvrABC system	IPR004602-<br>IPR004807-<br>IPR004791		
DinG	IPR006310		
RecA-MutS system	IPR016467-<br>IPR013765		
UvrD	IPR034739		
RecBCD pathway	IPR004586-<br>IPR006697-<br>IPR006344		
RecFOR pathway	IPR003395-<br>IPR003717-<br>IPR023627		
ATP-dependent DNA ligase	IPR012308		
DNA Repair Base Excision (KU, LigD)	IPR012310		
DNA ligase	IPR020923		

Supplemental Table S10B. InterProScan search results for CDA DNA polymerase domains.

Database	Description	Start	End	E-value	InterPro entry
Pfam	5'-3' exonuclease, N-terminal resolvase-like domain	4	169	2.20E-52	IPR020046
Pfam	5'-3' exonuclease, C-terminal SAM fold	170	249	1.00E-25	IPR020045
Pfam	DNA polymerase family A	487	863	0	IPR001098
SUPERFAMILY	PIN domain-like	2	169	4.49E-55	IPR029060
SUPERFAMILY	5' to 3' exonuclease, C-terminal subdomain	170	283	8.97E-31	IPR036279
SUPERFAMILY	Ribonuclease H-like	312	455	6.22E-18	IPR012337
SUPERFAMILY	DNA/RNA polymerases	461	864	0	IPR043502

Supplemental Table S10C. Top 3 structural analogs of CDA DNA polymerase I in PDB identified by TM-align.

Target	PDB Hit	Uniprot KB title	TM-score[1]	RMSD[2]	IDEN[3]	Cov[4]
Full protein	6vdeA	DNA polymerase I from *Mycolicibacterium smegmatis*	0.942	0.54	0.388	0.944
	1l3sA	DNA polymerase I from *Geobacillus stearothermophilus*	0.601	2.45	0.442	0.634
	1d8yA	DNA polymerase I from *Escherichia coli*	0.597	2.87	0.404	0.637
Ribonuclease H-like domain	6vdeA	DNA polymerase I from *Mycolicibacterium smegmatis*	0.968	0.90	0.222	1.000
	1d8yA	DNA polymerase I from *Escherichia coli*	0.877	1.96	0.201	1.000
	5dktA	Plastid replication-repair enzyme from *Plasmodium falciparum*	0.875	1.73	0.218	0.986

[1] Ranking of proteins is based on TM-score of the structural alignment between the query structure and known structures in the PDB library.

[2] RMSDa is the RMSD between residues that are structurally aligned by TM-align.

[3] IDENa is the percentage sequence identity in the structurally aligned region.

[4] Cov represents the coverage of the alignment by TM-align and is equal to the number of structurally aligned residues divided by length of the query protein.

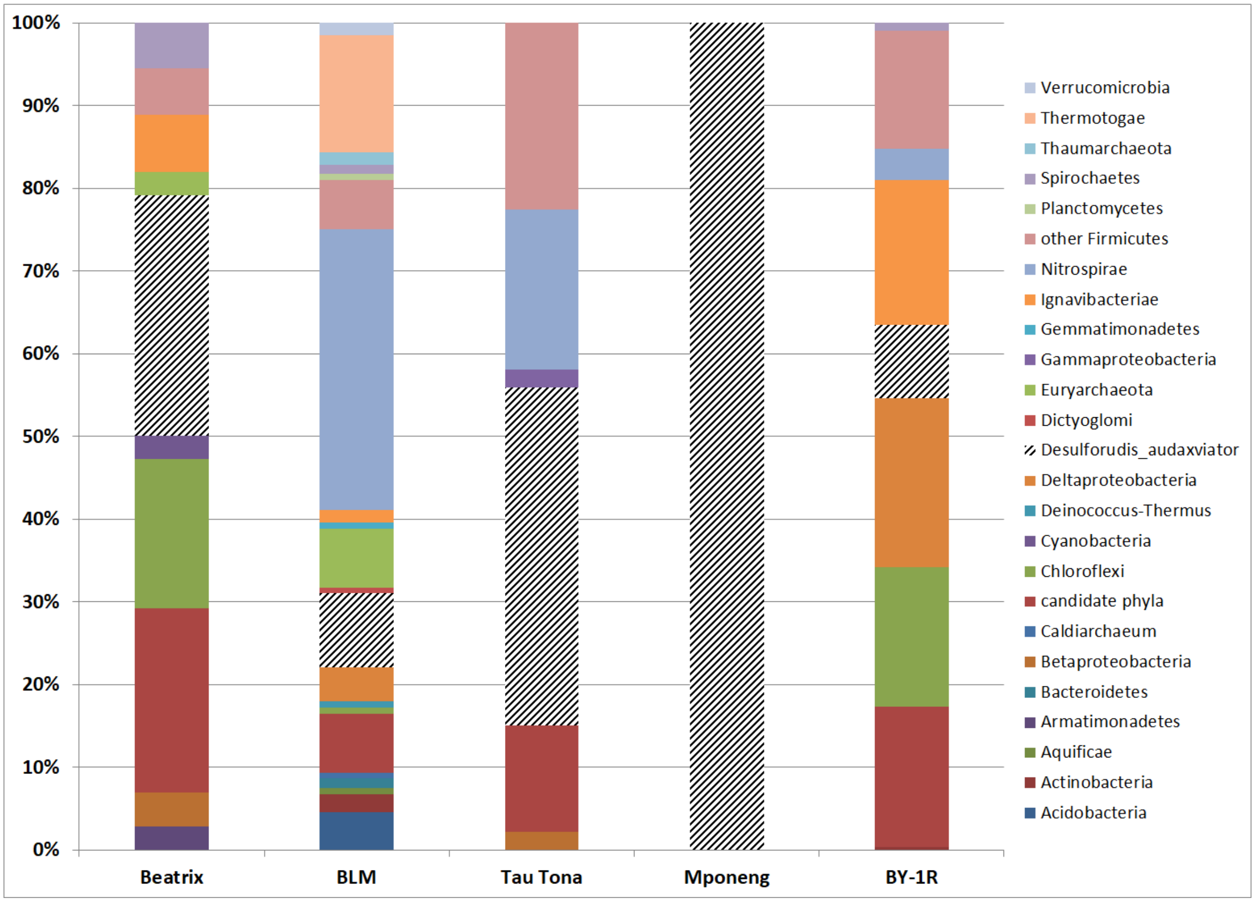

Supplemental Figure S1. Phylogenetic composition of SAGs obtained from the studied field samples. Taxa are reported at the phylum level, with the exception of the Proteobacteria, which are reported at the class level. The CDA (≥97% nucleotide identity of the 16S rRNA gene) are indicated with diagonal lines, in order to separate them from other Firmicute lineages.

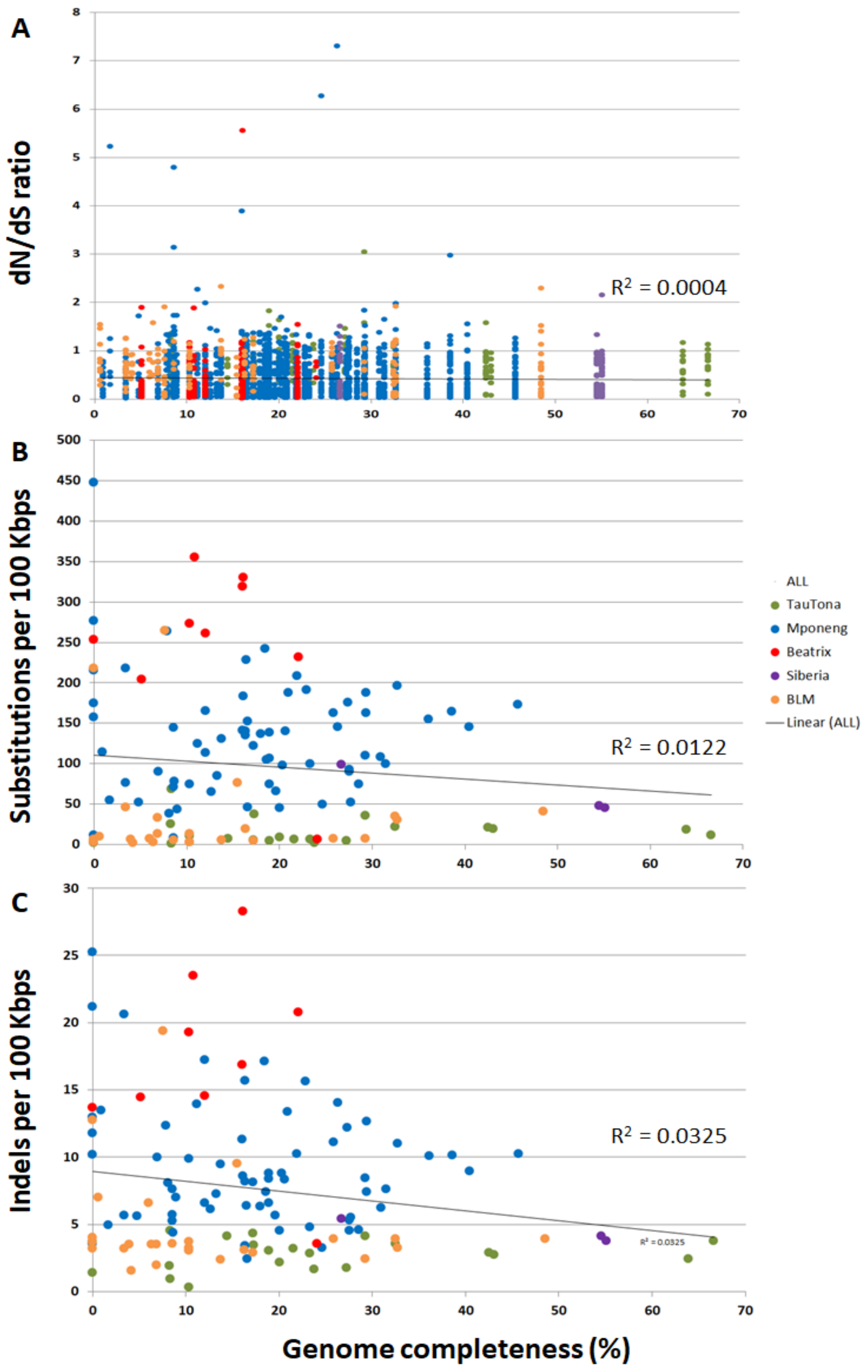

Supplemental Figure S2. Relationships between estimated SAG genome completion and nucleotide-level genetic differences from MAG MP104C: A) dN/dS ratio, B) substitutions per 100 kbps, and C) indels per 100 kbps.

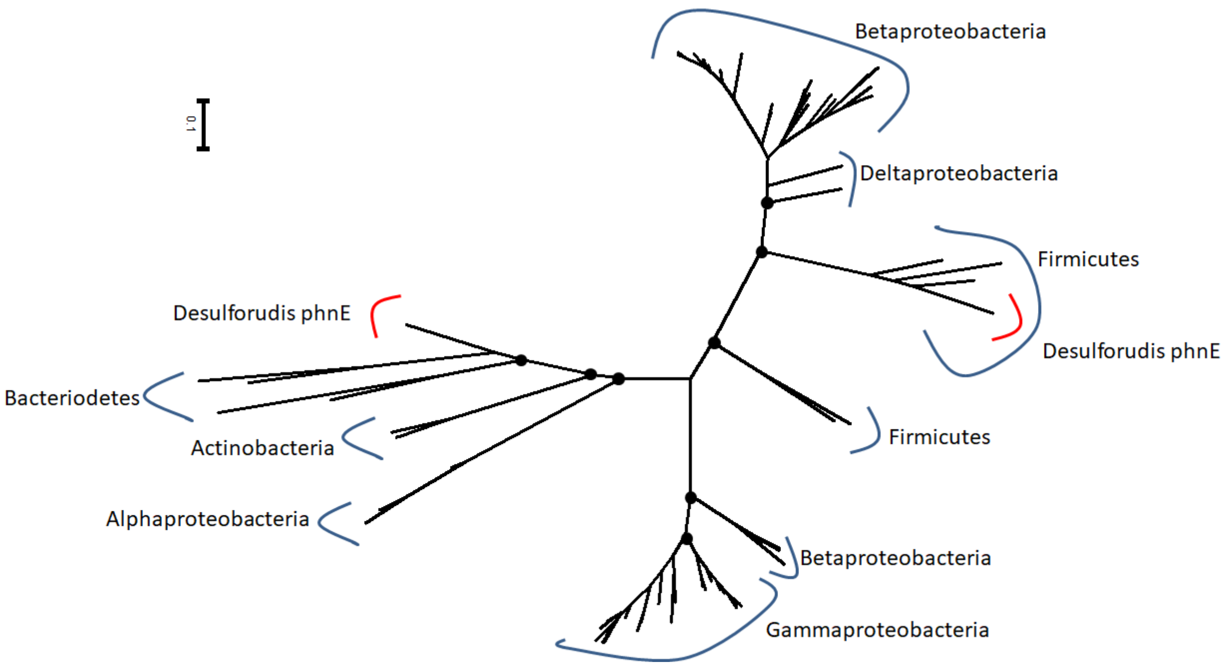

Supplemental Figure S3. Maximum likelihood phylogeny of the PhnE protein homologs found in both CDA SAGs and the complete MP104C genome from Mponeng, aligned with the top 100 non-redundant BLASTp hits in Genbank. Black circles represent major clades with strong bootstrap values (≥90). Scale bar indicates substitutions per site (0.1).

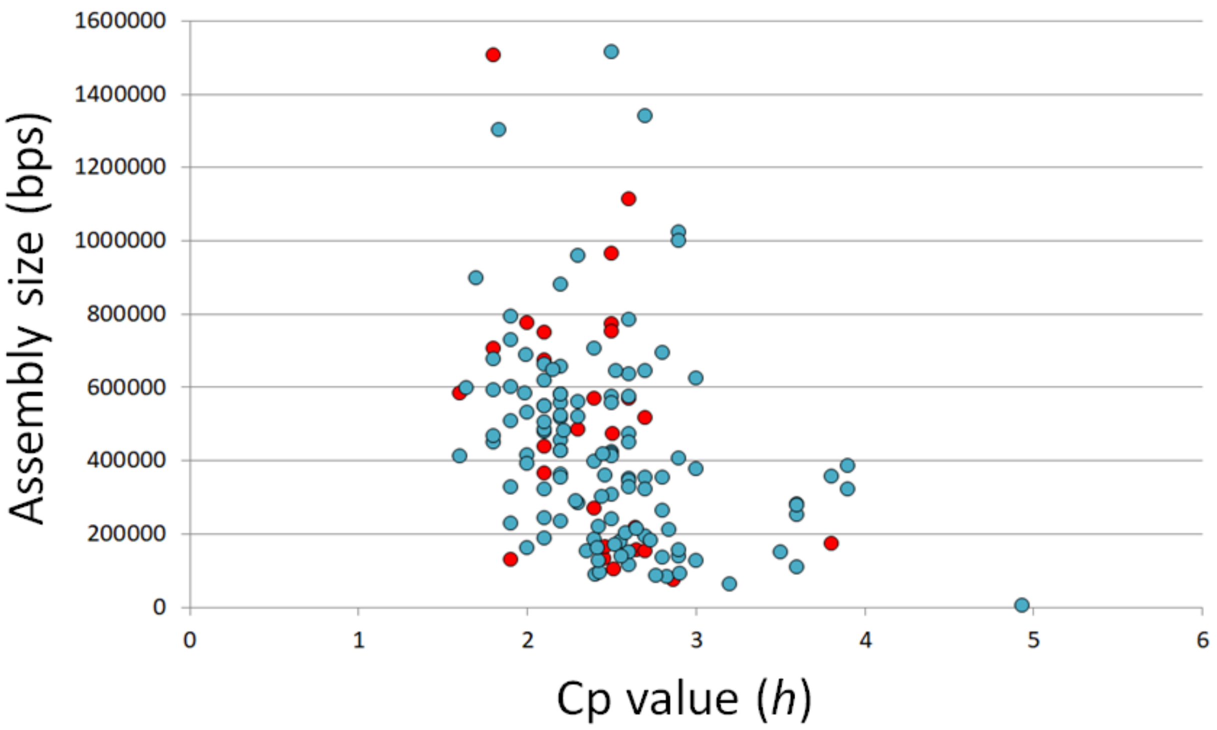

Supplemental Figure S4. Single cell whole genome amplification critical point (Cp) values plotted against genome assembly sizes. The Cp is estimated as the time required to reach the inflection point of the reaction's exponential phase [29]. CDA SAGs with putative viral contigs are indicated in red.

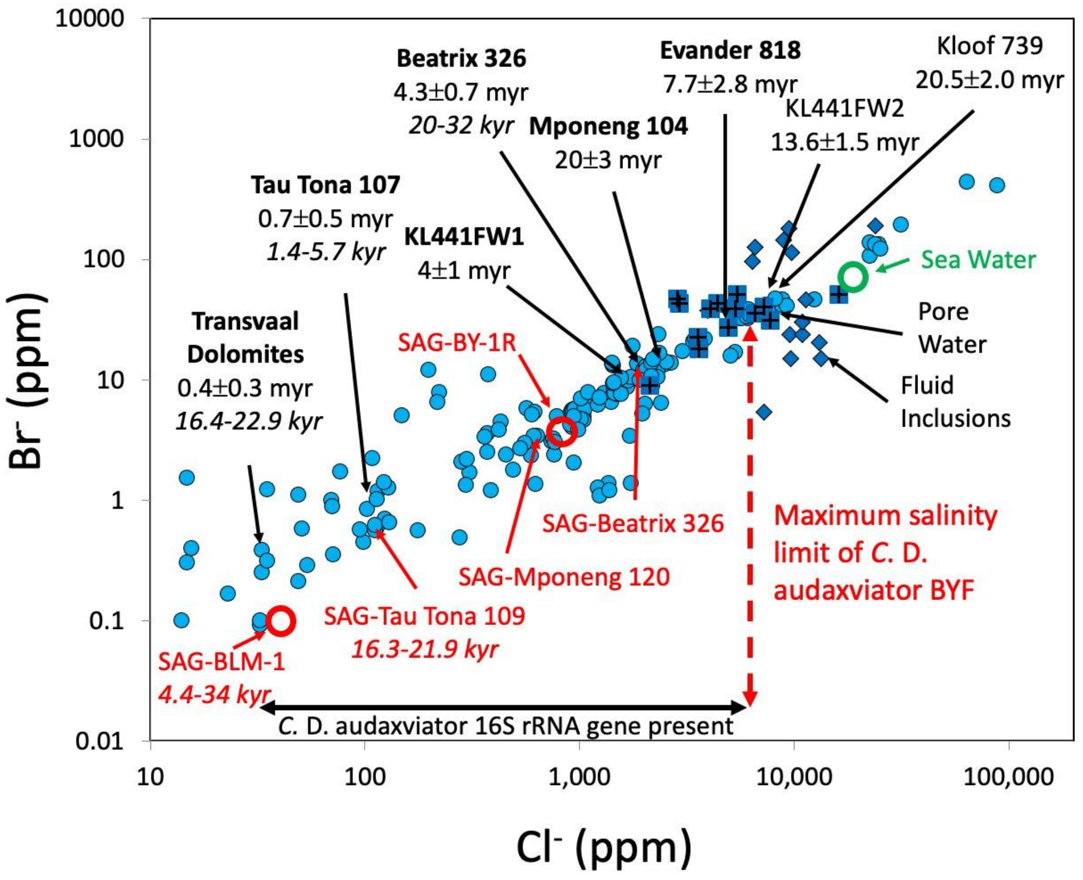

Supplemental Figure S5. Bromide versus chloride concentrations in ppm for fracture water samples (blue-filled circles), rock pore water (blue-filled squares with black crosses) and fluid inclusions (blue-filled diamonds) from the Witwatersrand Basin, South Africa [3, 4, 11, 31]. Those fracture water samples that have been dated by noble gas isotopic analyses [12, 14] (normal black font) and ^{14}C analyses of the dissolved inorganic carbon [13] (italics black font) are indicated by black arrows. Those same fracture water samples yielding CDA MP104C in 16S rRNA amplicon results are in bold black font. KL441FW2 and Kloof 739 do not contain CDA MP104C in 16S rRNA amplicon results. Fracture water samples yielding CDA SAGs are indicated in red arrows and font. The borehole water samples for SAGs Inyo-BLM 1 and BY-1R are shown with red circles, but no ages are implied by this comparison. The horizontal black double arrow indicates

the chlorinity range of fracture water samples where CDA was reported [4, 32]. The vertical red dashed double arrow represents the maximum salinity limit of the isolate CDA BYF recovered from borehole BY-1R in western Siberia. The average Br^- and Cl^- values of modern seawater is shown for comparison (green circle).

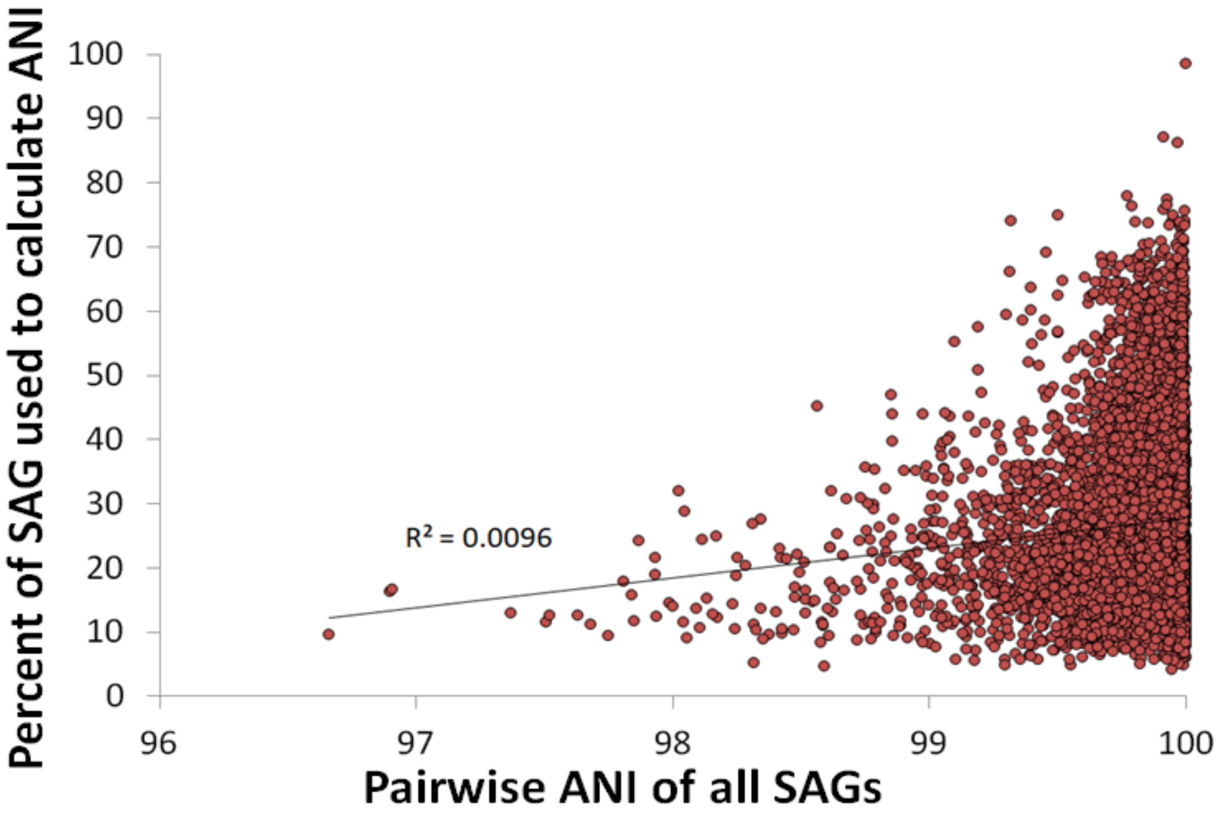

Supplemental Figure S6. Average nucleotide identity (ANI) calculated from all pairwise single amplified genome (SAG) combinations relative to the percent of each SAG genome used to calculate the ANI (i.e. the amount of genetic overlap for partial genome comparisons).

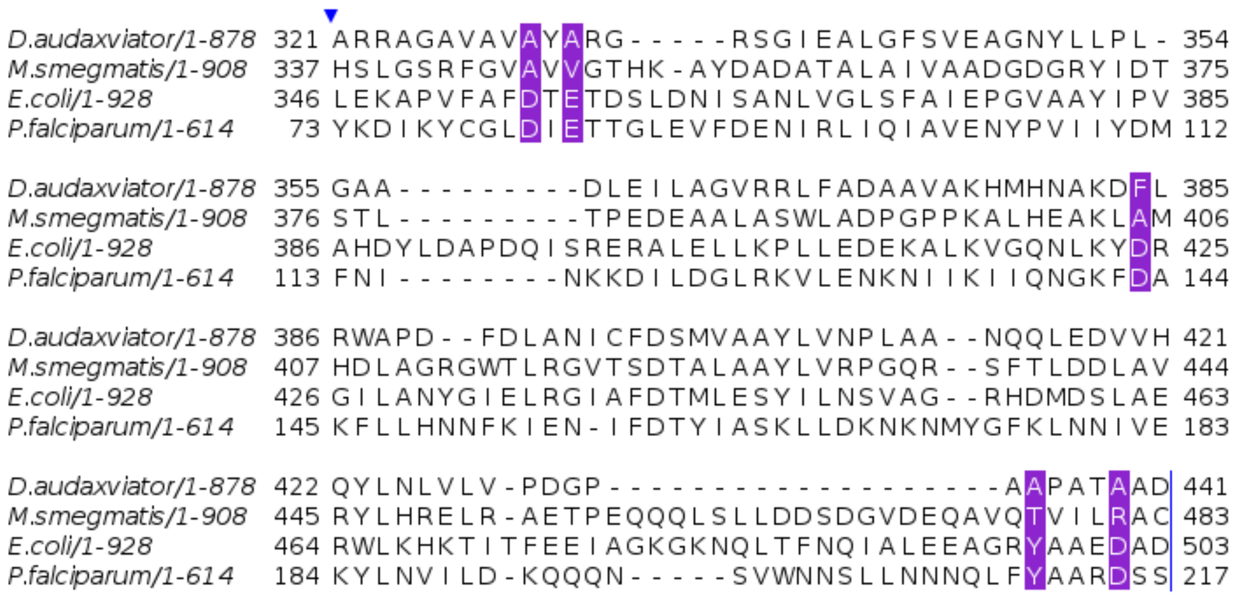

Supplemental Figure S7. Structure-based alignment of Ribonuclease H-like domain containing sequence fragments of CDA closest homologs. Purple color indicates catalytic residues of expected 3'-5' exonuclease domain.

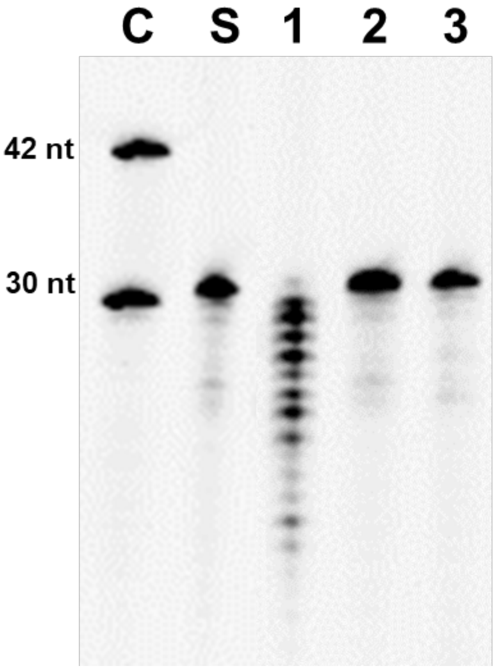

Supplemental Figure S8. Lack of *in vitro* 3'-5' proofreading activity in CDA polymerase I. Polyacrylamide gel electrophoresis image of 5’ phosphothiolated DNA-DNA oligoduplex hydrolysis reaction samples, showing 42 and 30 nt FAM labeled oligonucleotides control (C), DNA-DNA oligoduplex (S), SuperFi™ DNA polymerase hydrolysis reaction showing 3'-5' exonuclease activity (1), *Taq* DNA polymerase (lacking 3'-5' exonuclease activity) hydrolysis reaction (2), and CDA polymerase I hydrolysis reaction (3). 3'-5' exonuclease activity is not observed.

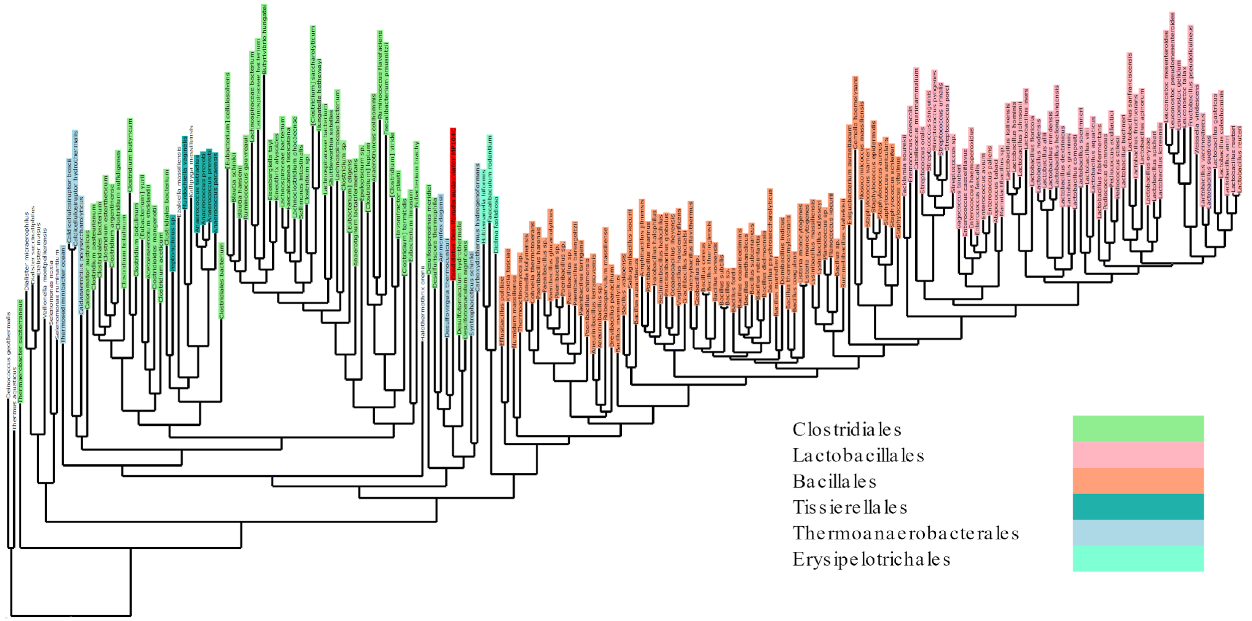

Supplemental Figure S9. *Firmicutes* DNA polymerase I protein phylogeny. The DNA polymerase I from CDA MP104C was analyzed together with 200 randomly selected orthologs. The red color denotes the polymerase from CDA. Other colors mark the most abundant orders as indicated in the legend. Phylogeny is anchored with polymerases I from T*hermus aquaticus* Y51MC23 and *Deinococcus geothermalis* DSM 11300.

Supplementary References

1. Frimmel H. Archaean atmospheric evolution: evidence from the Witwatersrand gold fields, South Africa. Earth-Science Reviews. 2005;70:1-46.
2. Lin LH, Hall J, Onstott TC, Gihring T, Lollar BS, Boice E, et al. Planktonic microbial communities associated with fracture-derived groundwater in a deep gold mine of South Africa. Geomicrobiology Journal. 2006b;23(6):475-97. doi: 10.1080/01490450600875829 PubMed PMID: ISI:000241195900010.
3. Lau CYM, Cameron C, Magnabosco C, Brown CT, Schilkey F, Grim S, et al. Phylogeny and phylogeography of functional genes shared among seven terrestrial subsurface metagenomes reveal N-cycling and microbial evolutionary relationships. Frontiers in Microbiology. 2014;5:531.
4. Magnabosco C, Tekere M, Lau MCY, Linage B, Kuloyo O, Erasmus M, et al. Comparisons of the composition and biogeographic distribution of the bacterial communities occupying South African thermal springs with those inhabiting deep subsurface fracture water. Frontiers in Microbiology. 2014;5:679-89.
5. Frank YA, Kadnikov VV, Lukina AP, Banks D, Beletsky AV, Mardanov AV, et al. Characterization and genome analysis of the first facultatively alkaliphilic Thermodesulfovibrio isolated from the deep terrestrial subsurface. Frontiers in Microbiology. 2016;7:2000. doi: 10.3389/fmicb.2016.02000. PubMed PMID: 28066337; PubMed Central PMCID: PMCPMC5165239.
6. Banks D, Frank YA, Kadnikov VV, Karnachuk OV, Watts M, Boyce A, Frengstad BS. Hydrochemical data report from sampling of two deep abandoned hydrocarbon exploration

wells: Byelii Yar and Parabel, Tomsk Oblast, Western Siberia, Russian Federation. NGU Report No. 2014.034. (Geological Survey of Norway, Trondheim, Norway, 2014).

7. Bredehoeft J, King M. Potential contaminant transport in the regional Carbonate Aquifer beneath Yucca Mountain, Nevada, USA. Hydrogeology Journal. 2010;18:775-89.
8. Winograd I. Interbasin groundwater flow in south central Nevada: A further comment on the discussion between Davisson et al. [1999a, 1999b] and Thomas [1999]. Water Resources Research. 2001;37:431-3.
9. Belcher W, Bedinger MS, Back JT, Sweetkind DS. Interbasin flow in the Great Basin with special reference to the southern Funeral Mountains and the source of Furnace Creek springs, Death Valley, California, U.S. Journal of Hydrology. 2009;369:30-43.
10. Mullin SW, Wanger G, Kruger BR, Sackett JD, Hamilton-Brehm SD, Bhartia R et al. Patterns of in situ mineral colonization by microorganisms in a ~60°C deep continental subsurface aquifer. Frontiers in Microbiology. 2020; 11:2573.
11. Onstott TC, Lin L-H, Davidson M, Mislowack B, Borcsik M, Hall J, et al. The origin and age of biogeochemical trends in deep fracture water of the Witwatersrand Basin, South Africa. Geomicrobiology Journal. 2006;23:369-414. doi: 10.1080/01490450600875688.
12. Lippmann J, Stute M, Torgersen T, Moser DP, Hall J, Lin L, et al. Dating ultra-deep mine waters with noble gases and ^{36}Cl, Witwatersrand Basin, South Africa. Geochim Cosmochim Acta. 2003;67: 4597-619. doi: 10.1016/S0016-7037(03)00414-9.
13. Simkus DN, Slater GF, Lollar BS, Wilkie K, Kieft TL, Magnabosco C, et al. Variations in microbial carbon sources and cycling in the deep continental subsurface. Geochim Cosmochim Acta. 2015;173:264–83.

14. Heard AW, Warr O, Borgonie G, Linage B, Kuloyo O, Magnabosco C, et al. Origins and ages of fracture fluids in the South African Crust. Chemical Geology. 2018;493:379-95.

15. Lin LH, Wang P-L, Rumble D, Lippmann-Pipke J, Boice E, Pratt LM, et al. Long term biosustainability in a high energy, low diversity crustal biome. Science. 2006b;314:479-82. doi: 10.1126/science.1127376.

16. Chivian D, Brodie EL, Alm EJ, Culley DE, Dehal PS, DeSantis TZ, et al. Environmental genomics reveals a single-species ecosystem deep within earth. Science. 2008;322(5899):275-8.

17. Hershey R, Mizell S, Earman S. Chemical and physical characteristics of springs discharging from regional flow systems of the carbonate-rock province of the Great Basin, western United States. Hydrogeology Journal. 2010;18:1007-26.

18. Finn RD, Attwood TK, Babbitt PC, Bateman A, Bork P, Bridge AJ, et al. InterPro in 2017-beyond protein family and domain annotations. Nucleic Acids Res. 2017;45(D1):D190-D9. doi: 10.1093/nar/gkw1107. PubMed PMID: 27899635; PubMed Central PMCID: PMCPMC5210578.

19. Jones P, Binns D, Chang HY, Fraser M, Li W, McAnulla C, et al. InterProScan 5: genome-scale protein function classification. Bioinformatics. 2014;30(9):1236-40. doi: 10.1093/bioinformatics/btu031. PubMed PMID: 24451626; PubMed Central PMCID: PMCPMC3998142.

20. UniProt Consortium T. UniProt: the universal protein knowledgebase. Nucleic Acids Res. 2018;46(5):2699. doi: 10.1093/nar/gky092. PubMed PMID: 29425356; PubMed Central PMCID: PMCPMC5861450.

21. DeLano W. PyMOL: an open-source molecular graphics tool. Ccp4 Newsletter Protein Crystallography 2002;40(11).

22. Park Y, Choi H, Lee DS, Kim Y. Improvement of the 3'-5' exonuclease activity of Taq DNA polymerase by protein engineering in the active site. Mol Cells. 1997;7(3):419-24. PubMed PMID: 9264032.

23. Brautigam CA, Sun S, Piccirilli JA, Steitz TA. Structures of normal single-stranded DNA and deoxyribo-3'-S-phosphorothiolates bound to the 3'-5' exonucleolytic active site of DNA polymerase I from Escherichia coli. Biochemistry. 1999;38(2):696-704. doi: 10.1021/bi981537g. PubMed PMID: 9888810.

24. Milton ME, Choe JY, Honzatko RB, Nelson SW. Crystal structure of the Apicoplast DNA Polymerase from Plasmodium falciparum: the first look at a plastidic A-Family DNA Polymerase. Journal of Molecular Biology. 2016;428(20):3920-34. doi: 10.1016/j.jmb.2016.07.016. PubMed PMID: 27487482.

25. Dagert M, Ehrlich S. Prolonged incubation in calcium chloride improves the competence of *Escherichia coli* cells. Gene. 1979;6:23-8.

26. Kriventseva EV, Kuznetsov D, Tegenfeldt F, Manni M, Dias R, Simao FA, et al. OrthoDB v10: sampling the diversity of animal, plant, fungal, protist, bacterial and viral genomes for evolutionary and functional annotations of orthologs. Nucleic Acids Res. 2019;47(D1):D807-D11. doi: 10.1093/nar/gky1053. PubMed PMID: 30395283; PubMed Central PMCID: PMCPMC6323947.

27. Chang JM, Di Tommaso P, Taly JF, Notredame C. Accurate multiple sequence alignment of transmembrane proteins with PSI-Coffee. BMC Bioinformatics. 2012;13 Suppl 4:S1. doi:

10.1186/1471-2105-13-S4-S1. PubMed PMID: 22536955; PubMed Central PMCID: PMCPMC3303701.

28. Kozlov AM, Darriba D, Flouri T, Morel B, Stamatakis A. RAxML-NG: A fast, scalable, and user-friendly tool for maximum likelihood phylogenetic inference. Bioinformatics. 2019. doi: 10.1093/bioinformatics/btz305. PubMed PMID: 31070718.

29. Lau MCY, Kieft TL, Kuloyo O, Linage B, Heerden Ev, Lindsay MR, et al. Deep-subsurface community dependent on syntrophy is dominated by sulfur-driven autotrophic denitrifiers. Proceedings of the National Academy of Sciences USA. 2016;113:E7927–E36. doi: 10.1073/pnas.1612244113.

30. Stepanauskas R, Fergusson EA, Brown J, Poulton NJ, Tupper B, Labonte JM, et al. Improved genome recovery and integrated cell-size analyses of individual uncultured microbial cells and viral particles. Nature Communications. 2017;8(1):84. doi: 10.1038/s41467-017-00128-z. PubMed PMID: 28729688; PubMed Central PMCID: PMCPMC5519541.

31. Borgonie G, García-Moyano A, Litthauer D, Bert W, Bester A, Heerden Ev, et al. Nematoda from the terrestrial deep subsurface of South Africa. Nature. 2011;474:79-82. doi: 10.1038/nature09974.

32. Gihring TM, Moser DP, Lin L-H, Davidson M, Onstott TC, Morgan L, et al. The distribution of microbial taxa in the subsurface water of the Kalahari Shield, South Africa. Geomicrobiology Journal. 2006;23:415-30.

33. Trias R, Ménez B, le Campion P, Zivanovic Y, Lecourt L, et al. High reactivity of deep biota under anthropogenic CO_2 injection into basalt. Nature Communications. 2017;8:1-4.

34. Alfredsson HA, Oelkers EH, Hardarsson BS, Franzson H, Gunnlaugsson E, Gislason SR. The geology and water chemistry of the Hellisheidi, SW-Iceland carbon storage site. International Journal of Greenhouse Gas Control. 2013;12:399-418.

35. Moser, DP, Hamilton-Brehm, SD, Fisher, Jenny C, Bruckner et al. Radiochemically-supported microbial communities: a potential mechanism for biocolloid production of importance to actinide transport. (United States Department of Energy, 2014). https://doi.org/10.2172/1176791. https://www.osti.gov/servlets/purl/1176791.

36. Hamilton-Brehm SD, Stewart LE, Zavarin,M., Caldwell M, Lawson PA et al.., Thermoanaerosceptrum fracticalcis gen. nov. sp. nov., a novel fumarate-fermenting microorganism from a deep fractured carbonate aquifer of the US Great Basin. Frontiers in microbiology. 2019;10:2224-2230.

37. Bangerter R.M and Giblin MO. Recompletion report for BILBY. Nevada Environmental Restoration Project. (National Technical Information Service, Springfield, VA, 1998). https://doi.org/10.2172/631144. https://www.osti.gov/servlets/purl/631144.

38. Garber MS and Johnson RH. A Summary of lithologic data, aquifer tests, and construction of hydraulic test well U-3cn#5, Nevada Test Site. Denver, CO. (United States Department of the Interior, Geological Survey, 1967).

39. Moser, DP, Bruckner J, Fisher J, Czerwinski K, Russell CE and Zavarin M. Characterization of microbial communities in subsurface nuclear blast cavities of the Nevada Test Site. (United States Department of Energy, 2010). https://doi.org/10.2172/1010281. https://www.osti.gov/servlets/purl/1010281.

40. Ege, JR, Carroll RD, Magner JE, Cunningham DR. US Geological Survey investigations in the U12n. 03 drift, Rainier Mesa, Area 12, Nevada Test Site, Nevada. (USGS Report USGS-OFR-80-1074, 1980).

41. NSTec Environmental Management. Completion Report for Well ER-EC-11 Corrective Action Units 101 and 102: Central and Western Pahute Mesa". (United States Department of Energy, 2010). https://doi.org/10.2172/1003755. https://www.osti.gov/servlets/purl/1003755.

42. NSTec Environmental Management. Completion Report for Well ER-EC-12 Corrective Action Units 101 and 102: Central and Western Pahute Mesa". (United States Department of Energy, 2011). https://doi.org/10.2172/1013015. https://www.osti.gov/servlets/purl/1013015.

43. Navarro, Inc. Pahute Mesa Well Development and Testing Analyses for Wells ER-EC-12 and ER-EC-13, Nevada National Security Site, Nye County, Nevada, Revision 0". (United States Department of Energy, 2018). https://doi.org/10.2172/1468445. https://www.osti.gov/servlets/purl/1468445.

44. NSTec Environmental Management. Completion Report for Well ER-EC-13 Corrective Action Units 101 and 102: Central and Western Pahute Mesa". (United States Department of Energy, 2011). https://doi.org/10.2172/1015229. https://www.osti.gov/servlets/purl/1015229.

45. Matsushita M, Ishikawa S, Magara K, Sato Y, Kimura, H. The potential for CH_4 production by syntrophic microbial communities in diverse deep aquifers associated with an accretionary prism and its overlying sedimentary layers. Microbes and Environments. 2020; 35:ME19103.

46. Matsushita M, Ishikawa S, Magara K, Sato Y, Kimura H. The potential for CH_4 production by syntrophic microbial communities in diverse deep aquifers associated with an

accretionary prism and its overlying sedimentary layers. Microbes and environments. 2020; 35:ME19103.

47. An D, Caffrey SM, Soh J, Agrawal A, Brown D, Budwill K, et al. Metagenomics of hydrocarbon resource environments indicates aerobic taxa and genes to be unexpectedly common. Environ Sci Technol. 2013;47:10708–17.
48. Trembath-Reichert E, Case DH, and Orphan VJ. Characterization of microbial associations with methanotrophic archaea and sulfate-reducing bacteria through statistical comparison of nested Magneto-FISH enrichments. PeerJ, 2016;4:e1913.
49. Teske A, McKay LJ, Ravelo AC, Aiello I, Mortera C, Núñez-Useche F et al. Characteristics and Evolution of sill-driven off-axis hydrothermalism in Guaymas Basin – the Ringvent site. Scientific Reports. 2019; 9:13847.
